# RNA Triple Helices: From Structures and Mechanisms to Therapeutic Targets

**DOI:** 10.7150/ijbs.132736

**Published:** 2026-08-21

**Authors:** Yiqi Ding, Zhechao Wang, Zimo Wu, Benyuan Cao, Haiyang Dong, Yongfeng Jin

**Affiliations:** 1 College of Life Sciences, Zhejiang University, Hangzhou, Zhejiang, ZJ310058, China; 2 School of Life Sciences, Zhejiang Chinese Medical University, Hangzhou, Zhejiang PR, ZJ310053, China

**Keywords:** RNA triple helix, gene expression regulation, RNA stability, RNA-targeted therapeutics

## Abstract

Major-groove RNA triple helices are conserved tertiary structures formed when a third strand inserts into the major groove of a classic double helix either via Hoogsteen base pairing or reverse Hoogsteen base pairing. These structures are widely distributed in eukaryotic, prokaryotic, and viral RNAs. This paper systematically summarizes the structural classification, stability-influencing factors, and identification methods of RNA triple helices. A growing body of evidence indicates that RNA triple helices are extensively involved in diverse biological processes, including RNA stability regulation, translation regulation, riboswitch ligand recognition, transposition regulation, telomerase activity, and the assembly of the spliceosome catalytic core, as well as serving as scaffolds for molecular recruitment. Notably, dysregulation of RNA triple helices is closely associated with tumorigenesis, viral infections, and genetic diseases. Based on their structural and functional characteristics, multiple therapeutic strategies targeting RNA triple helices have been explored, such as small molecules and antisense oligonucleotides. Collectively, RNA triple helices represent a pivotal link between RNA structural biology and precision medicine, with promising potential as an important candidate target for future RNA structural drug development.

## Introduction

RNA molecules achieve their essential biological functions through hierarchical folding, progressively evolving from linear sequences into complex three-dimensional architectures. Among these, the RNA triple helix represents a unique structural motif within RNA tertiary structures. The RNA triple helix (also referred to as the RNA triplex) consists of three polynucleotide strands assembled through specific hydrogen bonding and stacking interactions 1. The most common structure is the intramolecular triplex, in which the third strand originates from another single-stranded region of the same RNA molecule—such as a bulge, internal loop, or hairpin loop—and engages with the major groove of the Watson-Crick duplex. This interaction is mediated by Hoogsteen or reverse Hoogsteen hydrogen bonds, allowing the third strand to bind within the major groove of the duplex and form base triples, thereby stabilizing the local RNA architecture 2, 3. Concurrently, intermolecular triplexes formed by three strands contributed by two or three separate RNA molecules represent another important class of RNA tertiary interactions. These intermolecular systems broaden the structural and regulatory repertoire of RNA triple helices and provide a conceptual basis for developing RNA-targeted therapeutic strategies.

**Figure 1:**
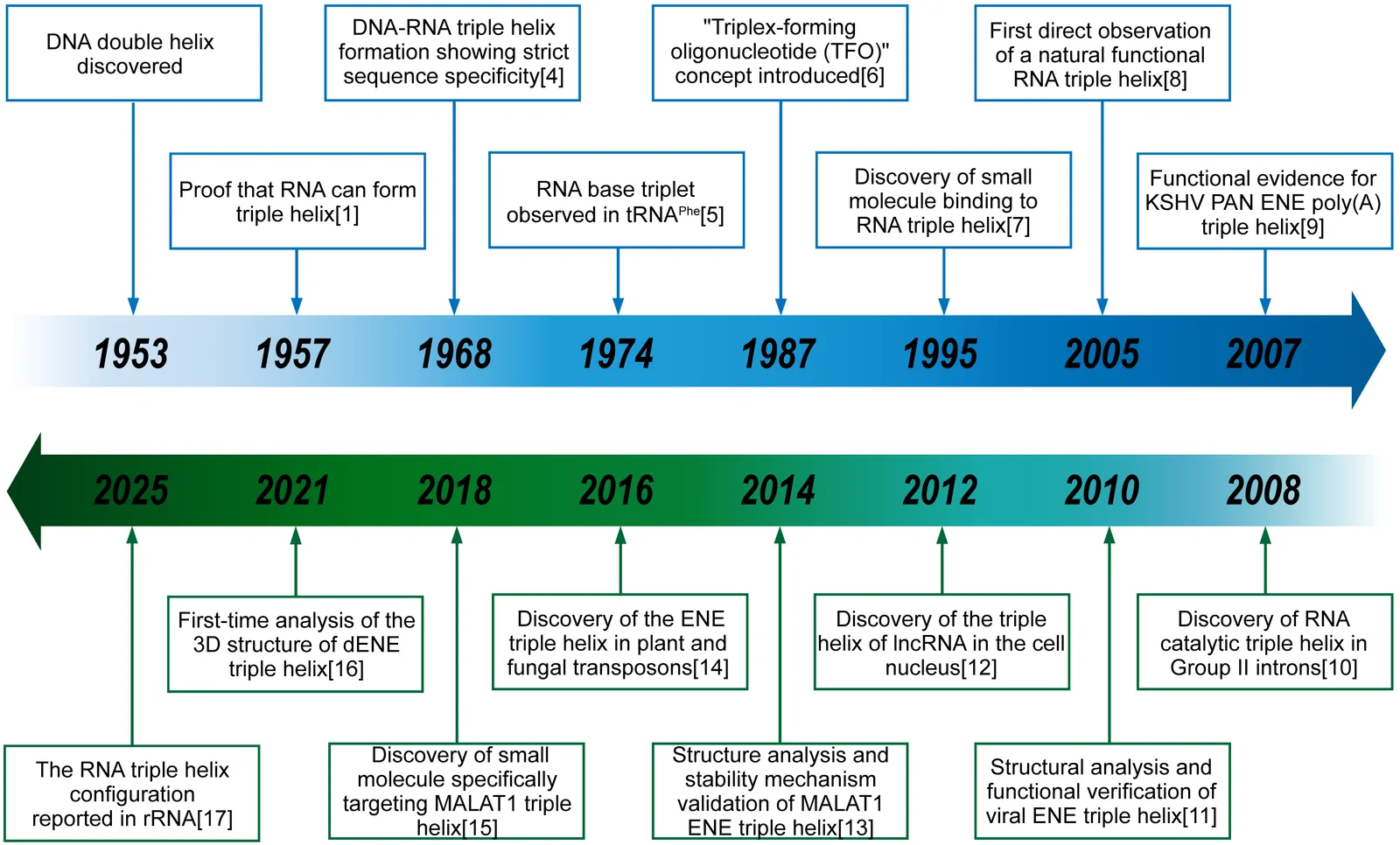
** Timeline of the discovery and development of RNA triple helices.** Over the past several decades, scientific research has progressed from the DNA double helix to the DNA-RNA triple helix and then to the RNA triple helix. The scope of research on the RNA triple helix has also expanded from telomerase RNA to eukaryotic spliceosome RNA, riboswitches, and nuclear expression elements. This field has advanced to exploring approaches such as targeting the RNA triple helix as a therapeutic target and other research directions.

The discovery and definition of the RNA triple helix have emerged from decades of cumulative research (Figure 1) 1, 4-17. Over the past decades, research has evolved from the DNA double helix to the DNA-RNA and RNA triple helices, expanding from telomerase RNA to spliceosomal RNA, riboswitches, and nuclear expression elements, while increasingly exploring RNA triple helices as therapeutic targets (Figure 1). In 1957, Felsenfeld, Davies, and Rich first demonstrated the existence of the triple helix *in vitro* 1. Using a system of synthetic poly(U) and poly(A) homopolymers, they discovered that a third poly(U) strand could specifically associate with the poly(A)·poly(U) duplex, leading them to infer the formation of a three-stranded complex. Although the detailed arrangement of the U·A-U triple helix had not yet been established at the time, subsequent studies confirmed this structural interpretation. This pioneering work established the foundation of nucleic acid triple helix research. Over the following decades, key breakthroughs were achieved, including the observation of naturally occurring continuous U·A-U major-groove triple helices 8 and the subsequent identification of RNA triple helices with clear biological functions 11. Advances in high-resolution structural biology—exemplified by the SAM (S-adenosylmethionine)-I riboswitch structure 18—and increasingly precise molecular techniques have driven the continuous discovery of RNA triple helices in viral, eukaryotic and prokaryotic RNAs (Figure 1). Figure 1 illustrates key time points and significant events in the study of RNA triple helices. Collectively, these findings demonstrate that triple helices are not rare structural anomalies, but rather widespread and functionally important RNA tertiary motifs (Figure 1). Accordingly, research in this field has evolved from the initial phase of structural identification to a new phase focused on functional characterization and the development of intervention strategies targeting triple helices.

This article reviews the structural and mechanistic basis of RNA triple helices, highlights their major biological functions, and discusses recent advances in therapeutic strategies targeting these structures. Furthermore, this paper outlines the key challenges and future directions in this rapidly developing field. Although several excellent reviews have summarized key advances in the biology of RNA triple helices 2, 3, 19, this paper places particular emphasis on the emerging field of extending structural and functional insights to disease intervention and therapeutic applications.

## Structure and Classification of RNA Triple Helix

### Identification and characterization of RNA triple helix

Techniques for identifying and characterizing RNA triple helices are continually advancing (Figure 1). The most definitive evidence comes from high-resolution three-dimensional structures, which can be resolved through nuclear magnetic resonance (NMR), X-ray crystallography, or single-particle cryo-electron microscopy (cryo-EM) 20-22. The ultraviolet (UV) thermal denaturation method monitors UV absorbance under a temperature gradient; RNAs containing triple helices often exhibit a biphasic melting profile in which the first transition reflects triple-helix dissociation and the second duplex unwinding, thereby enabling the analysis of thermodynamic parameters governing triple-helix formation and stability 23. Isothermal titration calorimetry (ITC) is also widely used to determine the thermodynamic characteristics of RNA triple helices, as heat changes can reveal binding stoichiometry and affinity constants, thereby obtaining data such as heat capacity 24. In a competition/indicator displacement assay, a signal-reporting probe is first bound to the RNA triple helix and test compounds are then added to monitor probe displacement as a readout of ligand binding 24. Currently, analytical methods for triple helices are becoming increasingly mature and diverse.

Since experimental discovery alone is insufficient to fully elucidate RNA triple-helical structures—particularly given that only a limited number of such structures have been identified in the human transcriptome—various software tools have been developed to search for and predict RNA triple helices. Because covariance models can capture conserved sequence and secondary-structure features, Infernal 1.1 can be used to detect homologous RNA regions that may preserve triple-helix-associated structural modules, even though it does not explicitly model the base triples themselves 25. More recently, several new computational frameworks have emerged to characterize long non-coding RNA (lncRNA)-associated RNA triple helices and their potential functions. For example, TRIPinRNA identifies RNA triple helices by scanning transcript sequences for patterns capable of forming canonical triples, as well as non-canonical hetero-purine/hetero-pyrimidine motifs, and predicts continuous triple-helix-forming regions across the full length of human transcripts, providing structural details including the Hoogsteen strand and potential gap positions 26; using this approach, the study identified plausible triplex-forming sequences in lncRNAs associated with X-chromosome inactivation, with selected candidates subsequently validated by biophysical assays. Researchers have developed an end-to-end computational process specifically for RNA-RNA-RNA triple helices. At the core of TripleMatcher is a dynamic-programming Matcher that searches candidates for unpaired third-strand segments aligned with continuous Watson-Crick-Franklin base-paired helices. When 3D structural information is available, these 2D candidates undergo further screening via 3DFilter, which uses C1′-C1′ distance thresholds to remove geometrically implausible cases. Finally, the ZoneCombiner integrates overlapping or nearby matches into non-overlapping candidate regions (“zones”), thereby highlighting broader RNA segments that may contain triple helices 27. TripleMatcher successfully localized all annotated triple-helix regions in eight experimentally validated RNAs, including telomerase RNAs and the lncRNAs metastasis-associated lung adenocarcinoma transcript 1 (MALAT1) and Kaposi's sarcoma-associated herpesvirus (KSHV) polyadenylated nuclear (PAN) RNA 27. Collectively, these advances are progressively establishing a comprehensive computational framework for discovering, annotating, and functionally interpreting RNA triple-helical structures across the human transcriptome. Nevertheless, these tools still share important limitations, including dependence on predefined sequence or secondary-structure patterns, incomplete representation of noncanonical and context-dependent triple interactions, sensitivity to the accuracy of input secondary or 3D structural models, and the need for experimental validation to distinguish true triple helices from computational candidates.

### Basic structure and classification of RNA triple helix

**Figure 2:**
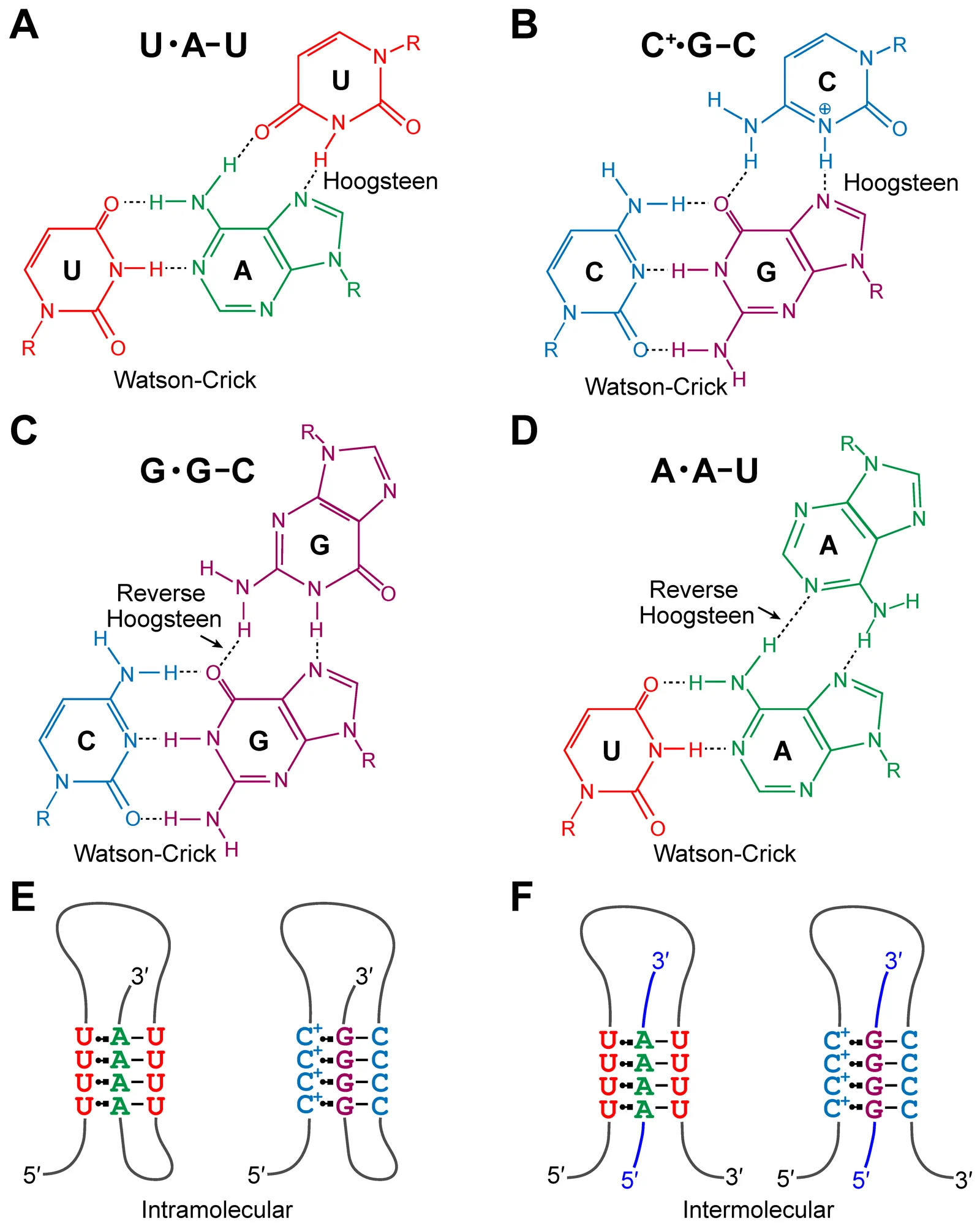
** Classification of RNA triple helix. (A, B)** The figures show U·A-U and C^+^·G-C base triples with Hoogsteen and Watson-Crick interactions, respectively. Hydrogen bonds are indicated by broken lines. Schematic diagrams of U·A-U and C^+^·G-C base triples are also shown on the right. Dashed lines represent hydrogen bonds. **(C, D)** The figure shows G·G-C and A·A-U base triples with the reverse Hoogsteen and Watson-Crick interactions.** (E, F)** RNA triple helices can be classified into intermolecular and intramolecular triple helices based on their molecular properties; if the three strands originate from the same transcript, they belong to intramolecular triple helices, whereas those originating from multiple transcripts (additional transcripts are indicated by blue lines) belong to intermolecular transcripts.

An RNA triple helix forms when a single-stranded oligonucleotide inserts itself into the major groove of a classical Watson-Crick duplex via sequence-specific hydrogen bonding, resulting in a characteristic triple-helical structure. On the basis of the composition and orientation of the third strand, as well as its mode of interaction with the underlying double helix, RNA triple helices can be classified into pyrimidine-type and purine-type triple helices. In a pyrimidine-type triple helix, the pyrimidine-rich third strand (U, C) binds parallel to the homopurine strand of the duplex through Hoogsteen interactions, forming stable U·A-U (Figure 2A) and C^+^·G-C triples (Figure 2B) (Where “·” represents Hoogsteen hydrogen bonds and “-” denotes Watson-Crick hydrogen bonds). Notably, this structure relies on the protonation of the N3 site in cytosine and its interaction with the N7 site in guanine, thereby forming the same number of hydrogen bonds as the Hoogsteen pair in the U·A-U triple 11, 12. In contrast, the third strand of purine-type triple helices is rich in purines (G, A), which bind to the purine strand of the duplex in an antiparallel orientation via reverse Hoogsteen interactions, predominantly forming triples such as G·G-C (Figure 2C) and A·A-U (Figure 2D) 28, 29. This classification highlights the decisive role of the third-strand sequence and orientation in determining the specificity and stability of triple-helix binding, providing a conceptual framework for understanding the rules governing triple-helix formation. RNA triple helices can also be categorized as intramolecular (Figure 2E) or intermolecular (Figure 2F) based on whether all three strands originate from the same transcript or from two or three separate RNA molecules. In addition, non-canonical triple-helix-like variants exist, in which the third strand inserts into the minor groove of a Watson-Crick duplex; and one of the most typical forms is the A-minor motif, in which adenine recognizes standard base pairs from the minor groove side, such as A·G-C base triples 9, 11, 13. In certain newly characterized riboswitches, the third RNA strand can insert into the minor groove and interact specifically with the duplex, thereby generating a local triple-helical structure 30. However, these triple helices are generally thermodynamically unstable. Therefore, this review will not elaborate further on the relevant content.

A growing number of RNA triple helices incorporate noncanonical base triples. For example, the group II intron in *Oceanobacillus iheyensis* contains a triple-helical structure composed of G·G-U and C·C-G base triples 10. Similarly, the bacterial SAM-II riboswitch has a U·U·A_SAM_ ligand-dependent base triple, in which the adenine moiety of SAM, rather than a regular RNA base, serves as the third base-like component in the interaction 31. In spliceosomal U2/U6 small nuclear RNAs (snRNAs) in *Schizosaccharomyces pombe* triple-helical formation involves base triples such as A·A-U, U·C-G, and G·G-C 29. The 7SK stem-loop 1, in a complex formed with a peptide fragment representing the RNA-binding domain of the human immunodeficiency virus trans-activator of transcription (HIV Tat) protein, possesses two consecutive U·A-U base triples; however, the protein recognizes its flanking C-G base pair 32, which differs from classical triples that can directly serve as protein-binding ligands. Moreover, researchers used the TRIPinRNA system to search for hetero-purine-pyrimidine intramolecular triple helices and discovered atypical A·U-A triples as well as transient motifs, such as UAA and AUU. These are less stable than classical triples and display higher structural plasticity and dynamic behavior 33. Together with high-resolution structural observations of other noncanonical triples in natural RNAs, including C·C-G and G·G-U in the group II intron 10, 34, U·U·A_SAM_ in the SAM-II riboswitch 31, and A·A-U, U·C-G, and G·G-C in spliceosomal U2/U6 snRNAs 29. Therefore, the design and engineering of RNA triple-helical structures should not be restricted to classical combinations such as U·A-U. Currently resolved RNA triple-helical structures are summarized in Table 1.

### Structural stability of the RNA triple helix

Having defined the structural features of RNA triple helices, it is essential to consider the factors that govern their formation and stability, as these ultimately reflect their thermodynamic and kinetic properties. A common method for measuring the stability of RNA triple helices is ultraviolet (UV) thermal denaturation, which works by monitoring the UV absorption spectrum of the sample under a temperature gradient to track its structural disruption process 23. Nucleotide composition is one of the primary determinants, particularly at Hoogsteen-interacting positions. Studies have shown that the identity of bases engaged in Hoogsteen (or reverse-Hoogsteen) pairing strongly influences thermodynamic stability, with pyrimidines generally providing greater stability than purines 35. Studies on the MALAT1 3′ end protective triple helix further demonstrate that pyrimidine·purine-pyrimidine arrangements exhibit superior stability 13. Moreover, C·G-C and U·G-C can also support highly stable RNA triple-helical formation 35. Importantly, sufficient thermodynamic stabilization is often required to sustain RNA function under physiological conditions, which provides useful guidance for the design of structured RNAs, including mRNA therapeutics. Specifically, RNA secondary structure can regulate antigen expression and the immunogenicity of mRNA vaccines through modulating RNA stability and translation efficiency 36, 37. In addition, RNA triple helices are dynamic rather than rigid structures, and their folding, assembly, and disassembly are influenced by multiple factors, which will be mentioned below. Accordingly, systems in which the third strand is released are also highly important.

In RNA triple helices, Hoogsteen base pairing often requires protonation of cytidine at the N3 position to form a hydrogen bond with guanine at the N7 position 12. Therefore, environmental pH is a critical determinant of triple-helical formation and stability. Melting temperature of the RNA triple helix decreases sharply with increasing pH, which is consistent with theoretical expectations 38. When the NaCl concentration was increased from 100 mM to 1 M, the melting temperature of the RNA trimer also decreased; this is presumed to be due to changes in salt concentration affecting the pKa value of N3 in cytosine, which in turn influences the formation of Hoogsteen hydrogen bonds in the RNA triple-helical structure 38. These factors also provide an important basis for assessing how RNA chemical modifications influence the stability of RNA triple-helical structures.

The peripheral structure of the RNA triple helix also plays an essential role in its stability. While studying the long non-coding MALAT1 RNA, which relies on the triple helix structure to maintain stability, researchers found that the peripheral double-stranded elements of its triple-helical structure aid in anchoring and stabilizing the RNA triple helix 39.

Some chemical ligands can significantly enhance the stability of RNA triple helices upon binding. For example, various isoquinoline alkaloids 40, the cationic dye methylene blue 41, and a novel ruthenium(II) polypyridine complex [Ru(dmb)_2_dppz-idzo]^2+^ 42 can all act as ligands to recognize and bind to RNA triples, enhancing their stability with strong affinity. Importantly, in addition to enhancing triplex stability, certain small molecules can also regulate and shift the equilibrium between RNA duplex and triplex conformations; for example, coralyne has been shown to induce both duplex-to-triplex and triplex-to-duplex conversion in poly(A)·poly(U)/poly(A)_2_·poly(U) systems in a temperature- and ligand/RNA ratio-dependent manner 43. Further details will be provided in the section of screening of small molecule drugs based on the RNA triple helix. The mechanism by which these ligand molecules recognize and bind to RNA triple helices provides valuable references and ideas for the future development of small molecules targeting RNA triple helices and the consequent expansion of intervention strategies for related diseases.

## Biological Functions of the RNA Triple Helix

### Enhancing RNA stability

**Figure 3:**
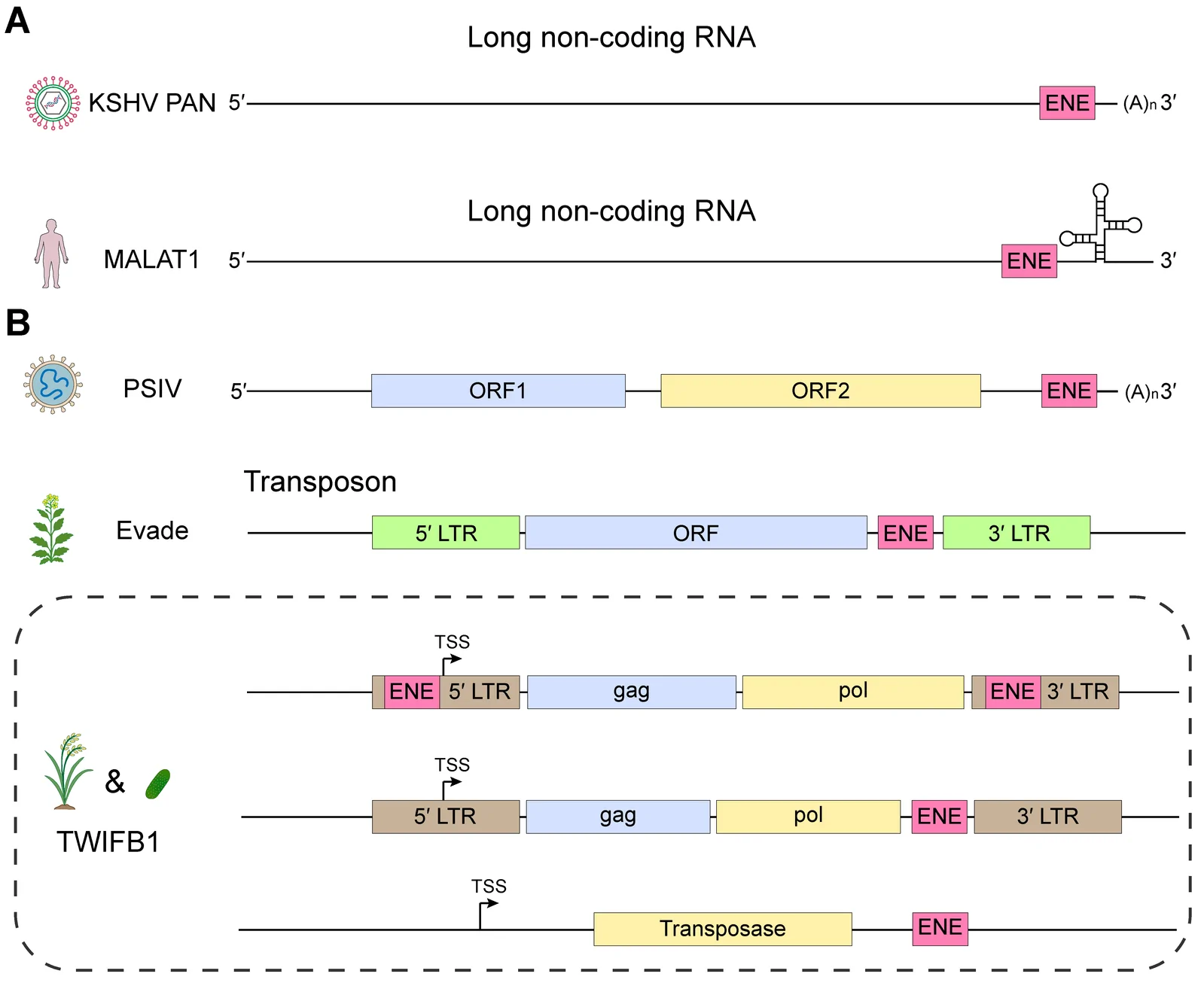
** Location of ENEs in genes and genomes. (A)** The most representative ENEs found in long non-coding RNAs are the ENE of viral PAN RNA and that of mammalian MALAT1. They are located near the 3′ end of their respective transcripts, used to protect the RNA from degradation by forming RNA triple helices, and the latter does not have the classic poly(A) tail 12, 13, 44, 119. **(B)** There are other ENEs that are located at the 3′ end immediately behind the ORF, such as the ENE of *Plautia stali* intestine virus (PSIV) and the ENE of TE in the Arabidopsis genome called Evade 23, 45. In the common transposable elements in plants and fungi, there are not only ENEs behind the 3′ LTR, but also ENEs embedded in the 5′ and 3′ LTR and ENEs behind the transposase gene, such as the ENE of the hAT DNA transposon TWIFB1 14. The genes (RNAs) and ENEs are not drawn to scale.

As an important conserved motif in RNA, the RNA triple helix plays a fundamental role in stabilizing the host RNA molecule and provides a platform for the establishment of diverse regulatory functions. The most representative example is the *cis*-acting element, expression and nuclear retention element (ENE), which contains characteristic RNA triple-helical structures. ENEs are found in diverse locations within genes and genomes, with the majority located near the 3′ end of transcripts (Figure 3). ENE was first discovered in KSHV PAN RNA 44. ENE typically consists of a U-rich internal loop flanked by short helical segments. This architecture enables the U-rich region to form a triple helix with the 3′-poly(A) tail, thereby spatially sequestering the poly(A) tail and preventing access by deadenylases. In this way, ENEs markedly enhance RNA stability 16. Since their initial discovery, ENEs have been identified in a wide range of cellular and viral RNAs 12, 14, 45. The most extensively studied ENE triple-helical structures include ENE from viral RNAs, ENE from mammalian oncogenic lncRNAs, and double-domain ENEs (dENEs) found in plants and fungi 12, 14, 44.

**Figure 4:**
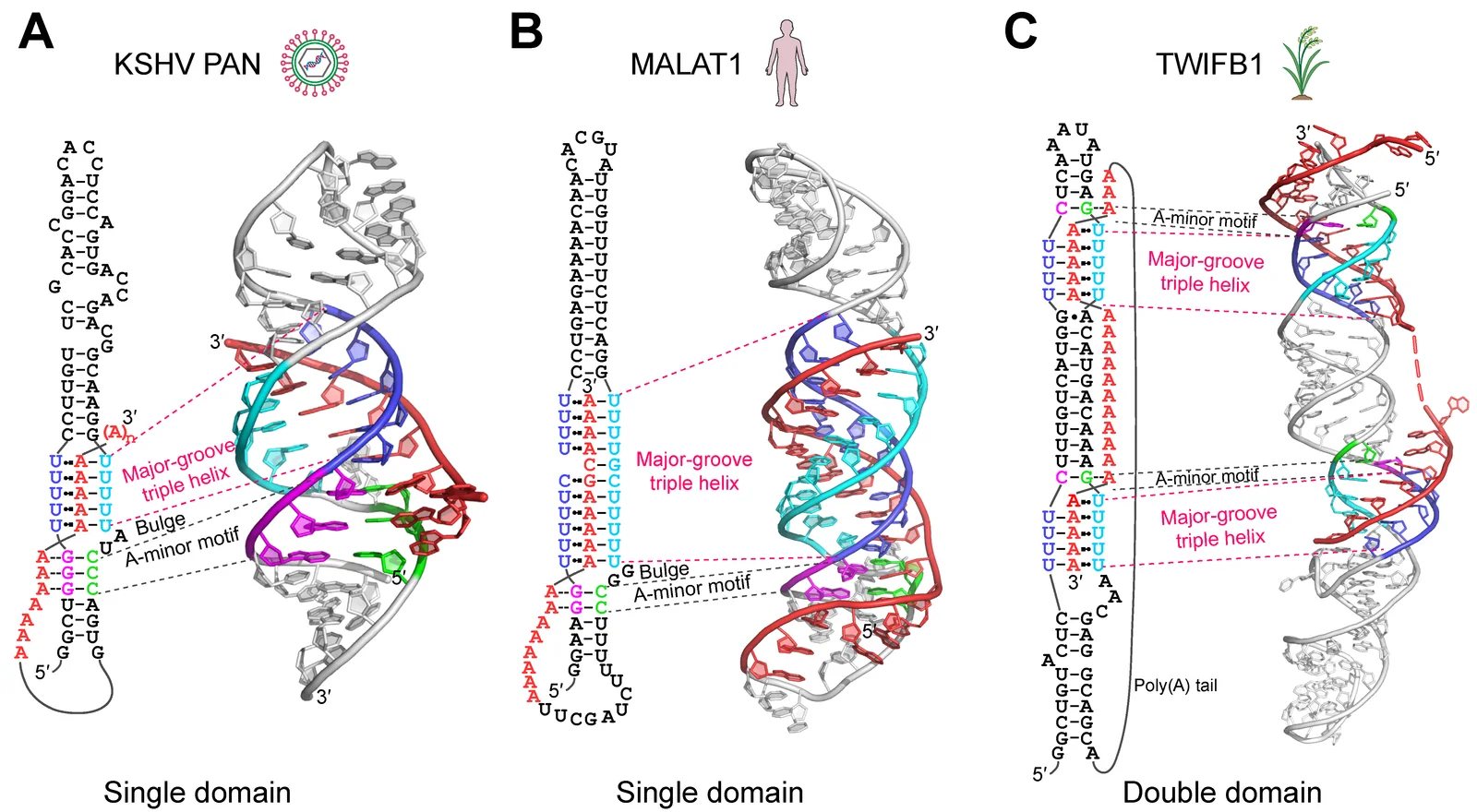
** The well-studied triple-helix of ENE elements primarily includes viral single-domain ENEs, blunt-ended ENEs of mammalian oncogenic lncRNAs, and dENEs from plants and fungi. (A)** Schematic diagram of the ENE element structure of KSHV PAN RNA, which belongs to the single-domain ENE category. The U-rich loop interacts with the poly(A) tail via hydrogen bonds, forming a U·A-U major-groove triple helix 11. **(B)** Schematic diagram of the 3′ end ENE element structure of MALAT1, which belongs to the blunt-ended ENE category. It forms a blunt end through the U-rich internal stem-loop and the downstream A-rich region, rather than terminating with a 3′-poly(A) tail 13. **(C)** Schematic diagram of the dENE structure in the rice hAT DNA transposon TWIFB1, which comprises the dENE, separated into two ENE motifs by a short double helix region. The right panel shows a schematic of the ENE triple helix structure 16. Structural models of RNA secondary and tertiary structures are shown in the supplementary files.

KSHV, the etiological agent of Kaposi's sarcoma, produces an abundant 1,077-nucleotide nuclear noncoding RNA known as PAN RNA. This transcript contains an approximately 79nt ENE that prevents degradation at its 3′ end, thereby stabilizing PAN RNA. The ENE folds into a U-rich internal loop flanked by short 5′ and 3′ helical stems. The U-rich loop engages with the 3′-poly(A) tail via simultaneous Watson-Crick-Franklin and Hoogsteen hydrogen bonds, forming canonical U·A-U base triples that constitute the core of the triple helix (Figure 4A) 46. This RNA triple-helix structure isolates the poly(A) tail to prevent RNA degradation by exonucleases such as deadenylases 11. In addition, ENE causes intron-less transcripts to be retained in the nucleus; however, its triple-helical structure only protects and maintains the transcript and does not necessarily enhance protein expression 47. These features establish the PAN ENE-mediated triple helix as a canonical model for poly(A) tail sequestration and viral nuclear RNA stabilization.

In human lncRNAs, the highly abundant and oncogenic MALAT1 and multiple endocrine neoplasia β (MENβ, also known as nuclear enriched abundant transcript 1 transcript variant 2, NEAT1_2) RNA contain triple helix elements analogous to the ENE found in PAN RNA. Unlike the PAN ENE, which interacts with the 3′-poly(A) tail, these lncRNA triple helices are formed by pairing a U-rich internal stem-loop with a downstream A-rich region, creating a triple helix possessing blunt ends. A canonical structural model, which is best characterized for MALAT1 and closely related in MENβ, comprises nine U·A-U base triples interrupted by a single C·G-C base triple and a C-G Watson-Crick base pair (Figure 4B) 12. Its mechanism of action also prevents degradation of the 3′ end by relevant enzymes 48. Functional dissection has been particularly well established for the MALAT1 3′-end triple-helix element, although much of this evidence has been obtained using exogenous reporter mRNAs or MALAT1-derived minimal constructs rather than full-length endogenous MALAT1. The same is true for studies on other types of RNA triples. Mutation-based β-globin reporter assays in MALAT1 further demonstrate that the precise arrangement of the internal U-rich loop and the downstream A-rich segment is critical for triplex stability and lncRNA activity 49. In summary, the MALAT1 and MENβ triple helices extend the ENE-mediated stabilization strategy to intron-less oncogenic lncRNAs. It is worth noting that through mass spectrometry identification, Clustered Regularly Interspaced Short Palindromic Repeats (CRISPR)-mediated deletion of the NEAT1_2 triple helix domain, and *in situ* hybridization, a recent study revealed that the triple helix maintains the integrity of nuclear paraspeckles, thereby mediating the nuclear localization and radiation-induced rapid activation of unphosphorylated microRNA 34a 50.

ENE-like *cis*-acting elements have also been identified in transcripts derived from plant and fungal transposable elements (TEs), termed dENEs 14. These elements are characterized by two ENE-like motifs separated by a short duplex region (Figure 4C). This structure, similar to the classic ENE domain, forms a protective triple helix or isolation structure with the 3′ end poly(A) tail, thereby blocking exonucleolytic attacks—including deadenylation—and promoting transcript stability 14. All structurally characterized dENEs harbor a highly conserved adenosine triad, and their flanking stem regions display strong sequence preferences 16. Unlike previously described ENEs, dENEs also possess a pocket motif at the downstream end of the triple helix. This pocket terminates the triple helix in a near blunt-end manner through steric hindrance, while simultaneously locking the terminal poly(A) adenosine within the major groove via hydrogen bonding, thereby shielding the 3′ end. By restricting accessibility to the 3′ end, this mechanism effectively inhibits degradation pathways such as deadenylation and reinforces RNA stability 46. Collectively, dENEs represent an evolutionarily adapted ENE variant that combines a conserved adenosine triad with a terminal pocket motif to maximize 3′ end protection and sustain RNA stability across diverse eukaryotic transcripts.

**Figure 5:**
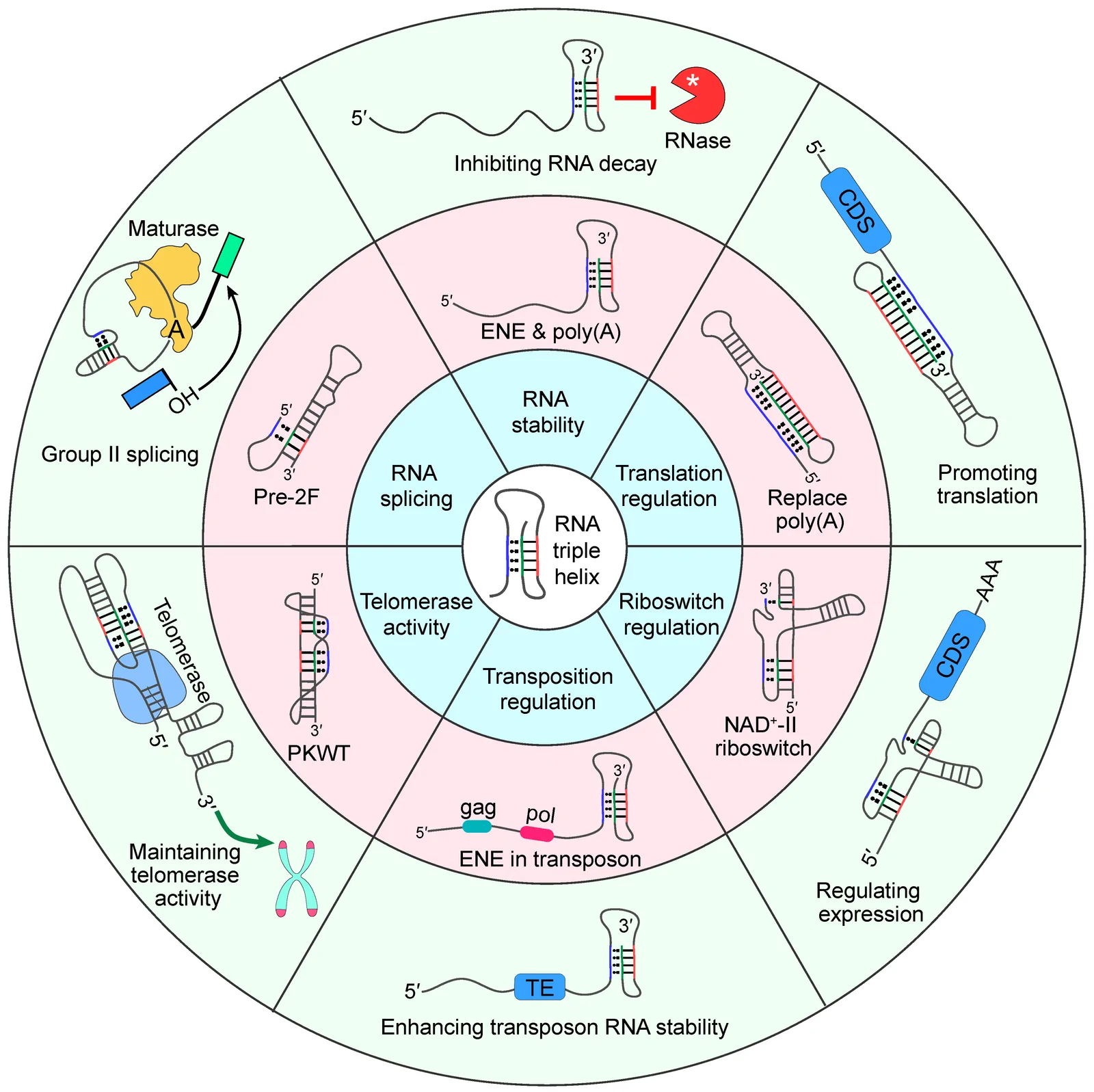
** Overview of the function of RNA triple helices.** The inner ring describes the biological functions of the RNA triple helix. The middle ring displays the structural features corresponding to the RNA triple helix performing different functions. The outer ring introduces the corresponding biological effects produced. Among these triple helices, group II introns, the eukaryotic spliceosome, and some types of riboswitches all possess antiparallel major-groove triple helices.

In addition to forming a triple helix with the 3′-poly(A) tail or an A-rich stretch to protect transcripts from exonuclease degradation, ENEs have also been shown to enhance the stability of nuclear transcripts, thereby increasing the mRNA levels of the corresponding genes; this phenomenon is particularly evident in intron-less mRNAs 14, 39. This is consistent with the mechanism by which ENE maintains viral RNA stability. These transposon-derived transcripts, having lost their introns (an adaptation to evade gene silencing), tend to reside longer in the nucleus and are thus more susceptible to nuclear RNA decay pathways 51. In this context, the presence of an ENE becomes especially important, as it confers nuclear stability and promotes the preferential accumulation of ENE-containing transcripts. It is worth noting that, besides RNA triple helices, evolutionarily conserved long-range RNA structures can also orchestrate complex gene regulation, as exemplified by the *Dscam1* exon 6 cluster, where multiplexed RNA secondary structures regulate the stochastic selection of variable exons in both temporal and spatial dimensions 52. Overall, RNA triple helices enhance host RNA stability by sequestering the 3′-poly(A) tail or A-rich region, thereby blocking deadenylases and other exonucleases from accessing the RNA end (Figure 5).

### Translation regulation

RNA triple helices can promote translation and, in some contexts, functionally replace the poly(A) tail (Figure 5). As early as in replication-dependent histone mRNAs, it was observed that other structures could replace the poly(A) tail 53. Researchers generated reporter constructs placing the 3′ region of MALAT1, which contains an ENE, downstream of the reporter gene to replace the poly(A) tail. They found that transcripts produced from these ENE-containing constructs exhibited protein expression levels comparable to those of transcripts bearing a normal poly(A) tail, suggesting that the ENE-mediated triple-helical formation stabilizes transcripts and sustains productive expression even without canonical polyadenylation 48. Transcripts with the poly(A) tail replaced by an ENE-mediated RNA triple helix can still be repressed by microRNAs (miRNAs), suggesting that triple-helix-based stabilization does not preclude interaction with standard post-transcriptional regulatory pathways. This supports the notion that ENE-driven triple helix formation can serve as an alternative 3′ end stabilization strategy without sacrificing normal regulatory inputs 48. Overexpression of PABPC1 in frog oocytes significantly enhanced the translation of short-tailed mRNAs, yet did not significantly promote the translation of mRNA at the 3′ end of the histone stem-loop or the MALAT1 triple helix, demonstrating that triple-helix-containing mRNAs can still maintain a certain level of translational output even in the absence of poly(A) tails, but are unable to bind to the poly(A)-binding protein in the cytoplasm to form closed-loop mRNAs 54. In most cases, enhanced protein yield arises indirectly through increased RNA stability rather than direct stimulation of the translational machinery. RNA triple helices restrict access of exonucleases and deadenylation complexes by forming a locally stable triple helix at the 3′ end, thereby prolonging mRNA half-life and expanding the pool of transcripts available for ribosome engagement, which in turn elevates translational output 46, 55. A recent study replaced the poly(A) tail of synthetic mRNA with the *Malat1* RNA triple helix and found that while it can support translation, its efficiency is far lower than that of the canonical poly(A) tail, suggesting that future optimization of triple helix sequence and folding is needed to enhance translational capacity 56. These findings establish the 3′ end RNA triple helix as a versatile, context-dependent regulatory module that, under certain conditions, can substitute for the poly(A) tail to maintain mRNA stability and translational competence, while still integrating into conventional post-transcriptional regulatory networks.

Major-groove base-triple interactions can also directly influence translation. A representative study showed that a major-groove triple helix within an mRNA stimulatory element enhances programmed ribosomal frameshifting by stabilizing an RNA structure that impedes ribosomal unwinding and promotes pausing at the slippery sequence 57. Disruption of the triplex reduced frameshifting efficiency, supporting the view that RNA triple-helical motifs play a direct role in translational recoding 57.

In the 25S rRNA of the large ribosomal subunit (LSU) from Entamoeba histolytica, a major-groove RNA triple helix has been identified, consisting of three U·A-U and one U·G-C base triples. This triple helix is located in domain VI (DVI), on the solvent-exposed side near the peptide exit tunnel (PET), marking the first reported instance of such a triplex conformation in rRNA 17. It is plausible that this triple helix, like its ENE counterpart, provides local protection against 3′ exonucleolytic or other rRNA processing or degradation activities, although this protective role remains to be experimentally confirmed. Furthermore, studies have found that ribosomal proteins and their extensions interact with the rRNA surface surrounding this triple helix, suggesting that the triple helix and its protein interactions help reinforce the outer surface of the LSU, thereby facilitating ribosome stability and subunit assembly, which in turn may contribute to more efficient translation.

### Riboswitch regulation

Riboswitches are a class of *cis*-regulatory RNA elements located in the non-coding region of mRNAs (usually the 5′ Untranslated Region, UTR) that can regulate gene expression by directly binding to small-molecule ligands (e.g., metabolites, ions, or coenzymes) 58, 59. Since the existence of riboswitches was first experimentally confirmed in 2002, over 50 different types have been identified, with widespread occurrence in bacteria and a small number found in archaea and eukaryotes 60, 61. Riboswitches act as receptors, capable of forming ligand-binding structures and executing regulatory commands, and they require no protein involvement to perform translationally regulated switch transitions 59.

A variety of riboswitches can form RNA triple helices in their ligand-bound states, including prequeuosine1 (preQ1)-I/preQ1-II/preQ1-III, guanidine III, SAM-V and SAM-II, cyclic-dimeric-guanosine monophosphate (c-di-GMP)-II, nicotinamide adenine dinucleotide (NAD^+^)-II riboswitch, Mn²^+^ riboswitch, adenine or purine riboswitch, and so on (Figure 5). Riboswitch triple helices are typically organized around a Watson-Crick duplex, with a third strand docking into either the major or minor groove. Such triple-helical motifs frequently occur in the principal closing helix of the aptamer domain (P1), the short local sub-helix adjacent to the ligand-binding core (P1.1), or in pseudoknot regions formed by long-range intramolecular pairing 30, 31, 62-65. Typically, the triple-helix structure of a riboswitch is directly formed by two U·A-U or A·G-C triple base pairs along with several nearby non-classical triple base pairs, which define the binding pocket for small molecules, thereby achieving highly selective recognition. Among the early identified riboswitch classes, SAM-V and SAM-II form major-groove triple helices, in which the third RNA strand docks into the major groove, with the SAM ligand positioned along the helical axis and stabilized by hydrogen-bonding interactions with surrounding bases at its ends 31, 64. One side of the guanidine III binding site has an opening that can accommodate ligand derivatives carrying small side chains or substituent groups, indicating both binding selectivity and a certain degree of structural plasticity 63. The preQ1-I/preQ1-II/preQ1-III classes adopt different folding patterns for the same ligand (preQ1), but all classes stabilize the RNA upon ligand binding, thereby altering expression platform accessibility 62, 66-68. C-di-GMP-II incorporates ligands as part of its three-stranded structure, suggesting that proteins may be involved in shaping the riboswitch conformation 68. These examples illustrate that riboswitch-associated RNA triple helices employ diverse architectural strategies to embed their ligands within three-stranded cores, thereby coupling highly selective small-molecule recognition with precise, structure-driven control of gene expression.

The NAD^+^-II riboswitch is a recently characterized RNA sensor capable of recognizing NAD^+^-related metabolites, with its core architecture stabilized by layered noncanonical interactions, including A·G-C base triples. This architecture broadens the structural repertoire of RNA triple interactions and illustrates that base triples can function not only as stabilizing elements of RNA folding, but also as direct contributors to metabolite recognition and riboswitch-mediated gene regulation 30, 65.

Furthermore, different riboswitch families employ distinct approaches to regulate RNA triple helices and are not confined to a single gene regulatory model. Some of the aforementioned riboswitch types can activate translation 69, 70, while others inhibit the translational process 30, 31, 62, 65, 69, 71-73. Still others can extend, pause, or terminate transcription 71, 72, 74. The ligand-binding triple helix regulatory mechanism of riboswitches exhibits remarkable flexibility and diversity. Future studies should employ high-resolution structural and kinetic analyses to determine the sequence of interactions between ligands and triple-helical structures. Characterizing these ligand interactions could serve as a reference for the design of RNA-targeting small-molecule drug design. Additionally, a systematic analysis should be conducted to investigate how RBPs influence the function of riboswitches under different strain conditions.

### Transposition regulation

Transposable elements, also known as transposons, are mobile DNA sequences found in nearly all eukaryotic genomes that can replicate and relocate within the host genome 75, 76. In eukaryotes, they are generally suppressed by transcriptional or post-transcriptional gene silencing mediated by small RNAs such as small interfering RNAs (siRNAs) and Piwi-interacting RNAs (piRNAs) 77, 78. In addition, RNA deadenylation removes the poly(A) tails and selectively degrades certain transposon transcripts that escape siRNA regulation, thereby suppressing new transposition events 79. However, some retrotransposons contain ENE elements, which form RNA triple helices to protect their transcripts from host-mediated decay, thereby modulating transposition (Figure 5) 13. Typically, ENE elements in transposons contain a U-rich internal loop (URIL) that binds to the 3′ poly(A) tail via concurrent Hoogsteen and Watson-Crick-Franklin interactions, while short flanking stems assemble the structure into a pocket-like architecture 16.

A notable example is the *Arabidopsis* Evade retrotransposon, which displays unusually strong transpositional activity. Sequence analysis revealed four U-rich repeat segments between its open reading frame (ORF) and the downstream long terminal repeat (LTR), capable of forming ENE-like stem-loop structures and supporting triple-helix formation 23. *In vitro* thermal denaturation experiments produced a characteristic biphasic melting profile, providing strong evidence for the presence of an RNA triple helix. Functional assays using a luciferase-based transient expression system with inducible mutagenesis further demonstrated that the ENE motif stabilizes the transcript and enhances gene expression. Finally, mutation experiments targeting the deadenylase CCR4a (an enzyme that shortens the 3′ end of retrotransposon RNAs) confirmed that the triple-helix-mediated protection is achieved by blocking CCR4a-dependent degradation 23. This indicates that the RNA triple helix structure serves as a vital survival strategy for transposable elements to maintain their activity within the host genome. These findings highlight that ENE-mediated RNA triple helices serve as a specialized adaptive mechanism through which retrotransposons evade host RNA decay pathways, thereby sustaining their transcript levels and ensuring their continued transpositional competence within the genome.

### Telomerase activity and telomere maintenance

Telomerase is a specialized ribonucleoprotein (RNP) that uses its RNA template to replicate chromosome ends, thereby solving the end-replication problem in eukaryotes 80. Its core components include telomerase reverse transcriptase (TERT), which catalyzes the synthesis of telomeric DNA repeats, and telomerase RNA (TR), which contains the template for telomeric DNA synthesis (referred to as hTR or TERC in humans, TER in ciliates, and TLC1 in yeast); in addition, several accessory factors are involved in its assembly, maturation, and localization 81. TR also contains several conserved sequences, among which the H-type pseudoknot structure is one of the most critical functional regions. A recent structural biology study has shown that the RNA triple helix found within this pseudoknot is a conserved structural element essential for telomerase catalytic activity (Figure 5) 82. Thus, the conserved triple helix embedded in the TR pseudoknot serves as a critical architectural hub that couples proper RNA folding with telomerase assembly and catalysis, thereby safeguarding telomere maintenance in eukaryotic cells.

By successively resolving the structures of human TR, *Kluyveromyces lactis* TR, several ciliate TERs, and *Tetrahymena thermophila* TER, it was found that this RNA triple helix is located in the stem of the pseudoknot forming a major-groove triplex composed of several consecutive U·A-U base triples; moreover, C^+^·G-C or U·G-C triples are present in yeast, whereas A^+^·G-C triples are present in *T. thermophila* 8, 22, 82, 83. C^+^ and A^+^ denote protonated cytosine and protonated adenine, respectively. Mutational studies of human TR show that disrupting these triple interactions markedly reduces the thermal stability of the pseudoknot and leads to loss of telomerase activity; conversely, compensatory mutations that allow the formation of C^+^·G-C triples at low pH partially restore structural stability and enzymatic activity, demonstrating the critical role of the triple helix in telomerase catalysis 8. Similar experiments conducted in *K. lactis*, *T. thermophila*, *Saccharomyces cerevisiae*, and other species have yielded consistent results 82-85, indicating that precise structural arrangement is essential for telomerase. Comparison with archaeal H/ACA ribonucleoprotein complexes, which normally guide the pseudouridylation of target RNAs, suggest that intramolecular pairing and fold-back extension of an opposing RNA strand in telomerase RNA generates a triple helix similar to the substrate-bound state, thereby blocking pseudouridylation and protecting telomerase RNA from nonspecific modification 21. These experiments demonstrate that RNA triple helices are necessary and widely conserved for maintaining telomerase activity.

Single-molecule experiments, NMR, and cryo-EM studies have shown that in full-length TR without protein, the pseudoknot triple helix does not usually represent the dominant conformation due to the presence of competing folding pathways; only when protein subunits (such as TERT and H/ACA-related proteins) participate in assembly does the triple helix form and stabilize the active state, suggesting that the triple helix may facilitate correct pseudoknot folding and may directly contribute to the positioning and assembly of groups near the catalytic site 8, 82-87.

The function of the triple helix is not isolated; together with surrounding domains such as the CR4/5 domain of Japanese medaka telomerase and the H/ACA domain and auxiliary proteins, it forms an RNP network that jointly determines telomerase activity and its biogenesis pathway 8, 88, 89. Recent studies have revealed that an RNA chaperone, La-related protein 3 (LARP3), competes with triple helix formation. This protein recognizes long 3′-extended transcripts that fail to fold correctly into the triplex and mediates their degradation; conversely, the formation of the triplex prevents LARP3 binding, blocks its degradation pathway, and promotes hTR maturation, thus maintaining a dynamic balance between the two 90. When this balance is disrupted—for example, by LARP3 overexpression—triple helix formation is inhibited, telomerase activity is reduced, and telomeres become shorter; in contrast, LARP3 knockdown promotes the production of mature hTR and leads to telomere elongation 90, both of which highlight the central regulatory role of the triple helix in quality control and RNA maturation.

In addition, some special cases have been observed in telomerases from different organisms. In* K. lactis*, mutations in the triple helix led to truncated telomeric repeat synthesis, suggesting that the triple helix may affect template usage or the translocation process 84. However, in *S. cerevisiae*, TERT does not require a triple helix to associate with TR 85. Furthermore, studies on TR biogenesis indicate that the RNA triple helix participates in determining the fate of precursor TR transcripts, particularly 3′-extended immature TR molecules, as the extension of TR transcripts can form an RNA triple-helical structure and recruit processing-related proteins 89. These organism-specific observations suggest that the telomerase RNA triple helix not only exerts flexible, species-dependent control over template utilization and enzyme translocation but also contributes to TR biogenesis by shaping the precursor processing and maturation pathways.

### RNA splicing regulation

Pre-mRNAs in eukaryotic cells contain introns, which are noncoding RNA sequences. Eukaryotic pre-mRNA splicing is a two-step reaction involving the removal of introns and the ligation of exons. The chemical steps of this mechanism are highly similar to those of self-splicing group II introns, as both proceed through two sequential transesterification reactions 91. Group II introns are usually 400-800 nt in length and fold into a characteristic six-domain (DI-DVI) spoke-like architecture around a central hub 92. Both systems exhibit the formation of a catalytic triplex to perform catalysis: in group II introns, the catalytic triplex is located in Domain V (DV), whereas in the spliceosome, the catalytic core is an RNA-based catalytic center analogous to that of group II introns (Figure 5). At the evolutionary and structural levels, the RNA scaffold of group II introns is considered an ancestral model for the spliceosome RNA-protein complex. Although many of the extensive scaffolding functions once performed by intron RNA have been replaced by proteins, the strategy involving the catalytic triplex has been retained 91, 92. The shared reliance on a conserved catalytic triplex in both group II introns and the spliceosome underscores a deep evolutionary continuity; even as much of the surrounding structural framework has gradually been handed over to proteins, an ancient RNA-based catalytic strategy has been preserved.

As mentioned earlier, the core catalytic structure of group II introns is located in the DV region. This region contains a highly conserved A-G-C catalytic triad and a C-G-C motif, which, together with the J2/3 joining strand—the RNA segment connecting domains II and III—form a catalytic triple helix. In conjunction with the dinucleotide bulge of DV, this triple helix coordinates two catalytic Mg²^+^ ions (M1 and M2) and participates directly in catalysis 10, 93-96. Among these, M1 accurately locates 2′-OH and stabilizes the departing group. M2 assists the departing group and coordinates an oxygen atom at the breaking site, thereby activating the nucleophile 97, 98. Cryo-EM and X-ray analyses at 3.0-3.5 Å resolution reveal that the metal-triplex catalytic platform remains unchanged during both transesterification reactions, while the substrate is exchanged through oscillation of the D6 branch helix. D6, the branch-site-containing domain VI of the group II intron, is anchored in the first step by contact with the thumb subdomain and DNA-binding (DBD) domain of the maturase; in the second step, it contacts intron domain II (D2) via downward oscillation 99. Group II intron uses this mechanism of solely exchanging substrates while maintaining the catalytic platform to fix magnesium ions, thereby maintaining the catalytic geometric configuration and stability.

The spliceosome in eukaryotes is a highly dynamic molecular machine composed of small nuclear ribonucleoproteins (snRNPs; U1, U2, U4, U5, and U6) and their associated auxiliary factors. It catalyzes intron removal and exon ligation through two sequential transesterification reactions 100. The core catalytic triplex of the eukaryotic spliceosome resembles the active-site architecture of self-splicing group II introns. In particular, the terminal GA of the U6 snRNA ACAGAGA sequence forms a major-groove triple helix interaction with the U2/U6 helix Ib, a segment of the U2-U6 complementary duplex. Concurrently, the apex of the adjacent U6 internal stem-loop (ISL) contains an AGC triad, which establishes three consecutive base triples with the U2/U6 helix Ib in a one-to-one fashion 96, 99, 101. Using cryo-EM, crosslinking mass spectrometry, genetic experiments, and metal-rescue approaches, researchers have demonstrated that these RNA elements enable U6 snRNA to coordinate two catalytic Mg^2+^ ions, thereby stabilizing the spliceosome conformation and promoting cooperative metal-dependent catalysis during splicing 28, 29, 102-104. Unlike group II introns, the eukaryotic spliceosome relies on extensive protein interactions with the U2/U6 RNA core to maintain structural stability and enhance catalytic efficiency 102, 103. Among these proteins, Prp8 is the largest and most conserved component of the spliceosome. Its core structure, together with surrounding subunits, forms a positively charged catalytic cavity that encloses the triplex active center. This surface neutralizes the negatively charged RNA phosphate backbone, facilitates substrate positioning, and is highly conserved from Stentor coeruleus to humans 101, 105. Moreover, the N⁶-methyladenosine (m⁶A) modification of the ACAGAGA sequence in U6 snRNA stabilizes the docking of U6 to the 5′ splice site (5′ SS) and promotes substrate entry into the catalytic triplex active center 106. These studies elucidate how proteins enhance spliceosome stability through precise contacts with the RNA triple helix and propose new mechanisms for the recognition and regulation of RNA base triples.

In addition, cryo-EM analysis revealed that the U12/U6atac helix Ib in the U12 small spliceosome forms a catalytic triple helix together with G19, A20, and U46 of U6atac, which also coordinates two metal ions, thereby inheriting the core geometrical conformation of the catalytic triplex of the Group II intron 104. Compared to the main spliceosome, it lacks the 5′ stem-loop but possesses a unique 3′ stem-loop with tighter base pairing and a higher number of pairs, compensating for the missing helix II structure and reflecting the conservation of U12 introns. These features indicate that the U12-dependent minor spliceosome preserves the ancestral triplex-based catalytic core of group II introns while maintaining splice site recognition and catalytic functions through distinct architectural adjustments.

### Structural scaffold

RNA triple helices function as dynamic, structure-dependent protein-binding scaffolds that mediate selective protein recruitment and regulate RNA maturation and activity. RNA triple helices can bind to proteins. The RNA methyltransferase METTL16 mentioned above interacts with RNA triple helices the need for methylation, whereas chemical modification of the triple-helical structure markedly reduces its binding affinity for METTL16 107. This suggests that RNA triple helices act as structural scaffolds that provide specific platforms for the binding of certain functional proteins and recruit effector molecules, with protein recognition being determined jointly by the triple helix architecture and the local chemical environment 107, 108. The triple helix arranges specific base triples into a continuous groove that forms a hydrogen-bonding network and hydrophobic stacking steps complementary to the side strands of α-helices or β-strands, which explains why some RBPs (such as METTL16) are highly sensitive to the *in situ* chemical environment of the triple helix 55. As modular, structure-specific docking platforms, RNA triple helices translate local chemical signatures into selective protein recruitment and downstream regulatory outcomes.

In addition, in living cells, the triple helix within the hTR pseudoknot undergoes conformational changes before and after assembly, thereby conferring the structural flexibility required for it to function as a protein-recruiting scaffold. As mentioned earlier, LARP3, LARP7, and MePCE dynamically bind to and exchange positions on different conformations of the hTR triple helix during its early maturation, thereby influencing hTR localization and activity and highlighting the continuous role of hTR as protein recruitment platform throughout its maturation process 17, 90, 109. The conformational plasticity of the hTR triple helix endows the telomerase RNA with a dynamic scaffold function, orchestrating the stepwise recruitment and exchange of RNP assembly factors during telomerase biogenesis.

## Diseases and Therapeutic Applications

### Mechanisms of RNA triple-helix-related diseases

#### Cancer

LncRNAs such as MALAT1 and MENβ promote cancer initiation and progression through multiple molecular mechanisms. The RNA triple helix structures at their 3′ ends can sequester the poly(A) tail, thereby preventing the activation of cellular RNA surveillance and decay pathways; moreover, in the absence of a canonical poly(A) tail, these triple helices can functionally substitute for the poly(A) tail to maintain transcript stability, enhance intracellular accumulation, and, to some extent, promote translation 13, 46, 110.

Although MALAT1 and MENβ are strongly implicated in cancer, there is a lack of direct evidence demonstrating that their 3′-end triple helices play a functional role. The best-established function of these triple helices is to stabilize transcripts and promote their nuclear accumulation 13, 46, 48, 110. This structural support may facilitate the oncogenic activities of MALAT1 and MENβ by maintaining sufficient RNA abundance. However, most of the cancer-related phenotypes reported for MALAT1 or MENβ have been observed in the context of the full-length lncRNAs and their associated RNP networks, rather than being directly attributed to the triple-helix structure itself 111-114. Thus, the triple helix is more accurately viewed as a supporting structural element in the functional activity of oncogenic lncRNAs.

In human telomerase RNA (hTR/TERC), the triple-helix-containing pseudoknot domain is essential for proper RNA folding, ribonucleoprotein assembly, and telomerase catalytic activity 8, 83, 85. Dysfunction of this structural element can lead to profound pathological effects. On the one hand, the maintenance of the hTR triple helix supports telomerase activity, which is frequently reactivated in cancer cells and contributes to their ability for unlimited proliferative capacity by sustaining telomere length 115, 116. On the other hand, destabilizing mutations or structural defects in hTR impair telomerase assembly or function, leading to telomere shortening, stem cell exhaustion, premature aging phenotypes, and telomere biology disorders such as dyskeratosis congenita 117, 118. Thus, the hTR triple helix links RNA structural integrity to both tumorigenesis and aging through its central role in telomere homeostasis.

#### Viral infection

As mentioned earlier, human herpesvirus KSHV PAN RNA contains a poly(A) tail or an A-rich region at its 3′ end, which folds back into the major groove to form a stable triple helix domain known as the ENE. This structure seals the 3′ end, preventing exonuclease-mediated deadenylation and degradation. As a result, PAN RNA accumulates to very high levels within the cell nucleus, accelerating the transition from viral latency to the lytic phase 9, 44, 119, 120. The presence of the triple helix enhances lytic replication efficiency, promotes virion production, and thereby increases the likelihood of reinfection of target cells 119, 121, 122. In addition, viral RNAs such as PAN RNA can directly modulate host transcriptional programs and the expression of cytokines and chemokines, contributing to chronic local inflammation or immunosuppression 123. Importantly, KSHV tunes triplex stability by structurally modifying its RNA across its life cycle, with reduced stability during latency when shorter RNA persistence aids immune evasion 124. Thus, the ENE-mediated triple helix in PAN RNA functions as a structural rheostat for viral RNA stability, enabling KSHV to amplify lytic replication, reshape local immune and inflammatory responses, and flexibly toggle between latent persistence and productive infection.

Another important function of this class of ENE triple helix is to promote nuclear retention of PAN RNA and recruit host chromatin-modifying complexes, thereby establishing an epigenetic environment favorable for viral transcription and activating and enhancing the transcription of lytic genes 122. At the same time, several protruding bases within the triple helix confer conformational flexibility, enabling it to stabilize PAN RNA while maintaining sufficient adaptability for protein binding and recruitment 124. Furthermore, some long-term latent human viruses rely on triple-helical structures to maintain high levels of transcription, thereby contributing to chronic inflammation, immune evasion, and even tumor formation 11, 12, 120, 121, highlighting the convergent evolutionary significance of this structure in the regulation of viral RNA homeostasis. These findings indicate that ENE-like triple helices function as conserved viral RNA platforms that couple nuclear retention, chromatin remodeling, and RNA processing to sustain high-level viral gene expression; in long-term infections, they serve as a co-evolutionary strategy enabling immune evasion, chronic inflammation, and even oncogenic transformation, thereby controlling viral RNA homeostasis.

#### Genetic diseases

Mutations in genes that control the formation of RNA triple helices can disrupt or fail to form these triple helices, resulting in loss or gain of function and consequently triggering disease; such disorders are generally hereditary. The triple-helical structures present in telomerase are crucial for its catalytic activity and play an important role in the correct folding of specific regions of hTR and the maturation of precursor mRNAs. Therefore, if the genes that govern the formation of these triple helices are mutated, leading to mutations in the template/pseudoknot (t/PK) core region of hTR (such as the hTR n.96_97delCT mutation and mutations at the hTR 107/108 sites), the resulting disruption of the triple-helical structure decreases hTR stability, reduces telomerase catalytic activity, aggravates oxidative damage to telomeric DNA, accelerates telomere shortening and premature cellular aging, and triggers genetic diseases such as dyskeratosis congenita (DC), idiopathic pulmonary fibrosis (IPF), and aplastic anemia (AA) 8, 125.

Furthermore, the pathological consequences of hTR triple-helix disruption extend beyond the loss of catalytic efficiency alone. Studies have shown that the H/ACA complex can disrupt the precursor hTR triple helix, thereby permitting processing by RRP6 and PARN; meanwhile, LARP3 preferentially recognizes misfolded 3′-extended hTR precursors and promotes their turnover 89, 90. Notably, there is currently a lack of a sufficiently validated spectrum of recurrent human hereditary disease-causing mutations.

### Screening of small molecule drugs based on the RNA triple helix

**Figure 6:**
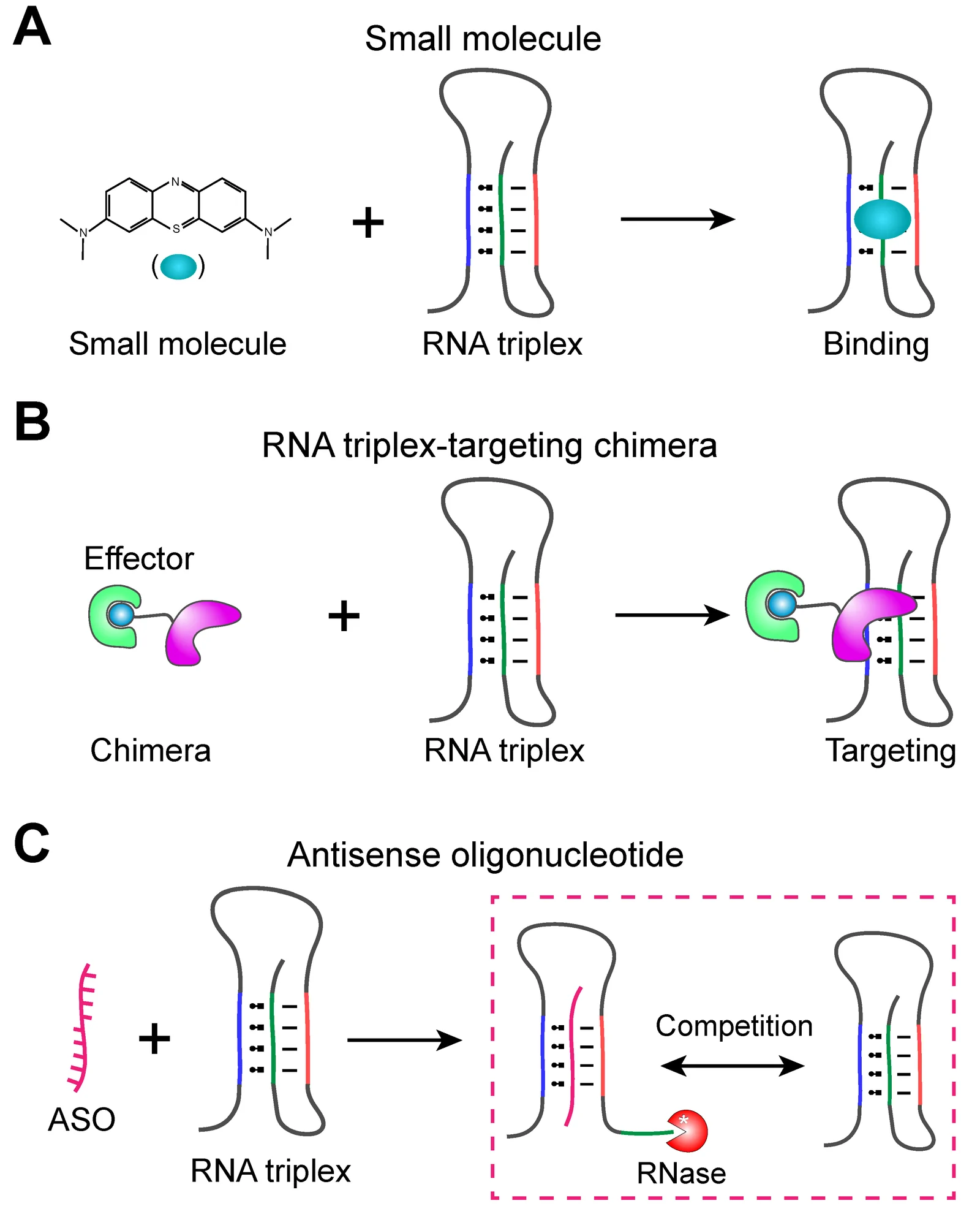
** RNA triple helix acts as therapeutic target. (A)** Small molecules directly target RNA triplexes and produce corresponding effects. **(B)** RNA triplex-targeting chimeras employ molecules that target RNA triplexes as probes and are coupled with effectors that selectively reduce harmful transcripts. **(C)** ASOs target regions of the U-rich main strand or A-rich complementary strand required for triplex formation, competitively displacing the natural third strand. Among these, small molecules (A) and ASOs (C) have been experimentally verified in RNA triple helices, while RNA triplex-targeting chimeras (B) are mainly derived from other RNA-targeted therapeutic strategies. A strategy similar to it, PINADs, has been experimentally verified in RNA triple helices.

The application of RNA triple helices as therapeutic targets for related diseases is still in its infancy, with several targeting strategies currently in development, among which research on small-molecule drugs based on RNA triple-helical structures has been relatively well-established (Figure 6A). A key challenge in designing small molecules that target RNA triple helices is ensuring their specific binding to RNA triple helices 126.

#### Cancer therapeutic targets

In cancers driven by lncRNAs such as MALAT1 and MENβ that harbor RNA triple helices, these triple-helix motifs enhance transcript stability by shielding the RNA from nuclease-mediated decay, making them highly attractive therapeutic targets. Notably, these lncRNAs do not acquire the canonical 3′-poly(A) tail. Instead, their precursor transcripts are processed by RNase P to generate a mature 3′ end, followed by subsequent processing to produce MALAT1-associated small cytoplasmic RNA (mascRNA) 127. During maturation, the ENE element containing a U·U base pair folds into an extended and conformationally restricted structural unit. However, the RNA remains vulnerable to exonucleolytic degradation at this stage, indicating that the triple helix is in a metastable state 110. This U·U base pair thus represents a pharmacological hotspot: rationally designed small molecules can recognize and disrupt this motif 128, 129, thereby impairing triple-helical formation and stability, and consequently reducing steady-state lncRNA accumulation and oncogenic activity. Wilusz et al. demonstrated by constructing expression plasmids that recapitulate the 3′ end processing of MALAT1 that the internal U-loop of the ENE and the short A-rich tract are essential for transcript stability 48. Accordingly, therapeutic strategies may aim to perturb this region through structural engineering, chemical modification, or small-molecule targeting, thereby disrupting triplex integrity and reducing RNA stability. For instance, incorporating 2′-O-methyl sugar modifications can destabilize the triple helix 130, rendering the RNA susceptible to nuclease-mediated degradation and thereby eliminating oncogenic lncRNAs. Another small-molecule-based approach involves inhibiting other cofactors that associate with the triple helix to block lncRNA function, such as METTL16, which interacts with the aforementioned ENE triple helix and represents a potential therapeutic target 107. A recent study integrated a structure-based drug discovery (SBDD) pipeline comprising molecular dynamics simulations, pocket dynamics analysis, ensemble docking, and multiple scoring functions to evaluate diminazene ligands targeting the MALAT1 triple helix, identified two potential binding sites, revealed that binding affinity is primarily driven by hydrogen-bonding and electrostatic interactions, yet highlighted the limited predictive accuracy of current computational workflows 131.

Multiple small molecules have emerged as potential therapeutics targeting RNA triple helices (Table 2). Since discovering ligands with sufficient specificity for RNA triple helices is essential for therapeutic development, high-throughput methods, such as small-molecule microarray (SMM) screening, have been employed for initial hit identification 132. In the case of the MALAT1 triple helix, Abulwerdi et al. identified two imidazole derivatives, compounds 5 and 16, that specifically bind this triple helix through SMM screening and validation in breast tumor organoids 133. These compounds reduced MALAT1 RNA levels by 54% in breast tumor models without affecting homologous triple-helix-forming RNAs, indicating that they possess a certain degree of selectivity and limited off-target effects 133. The natural flavonoid quercetin also exhibits similar activity 134. Several diminazene derivatives targeting the MALAT1 triple helix can achieve bidirectional modulation: they can either stabilize the triplex via hydrogen bonding and electrostatic interactions, reducing nuclease accessibility, or induce conformational rearrangements to expose the poly(A) tail, thereby promoting nuclease recognition 24. The aromatic heterocycle M5, based on a diazaindene scaffold, can disrupt the stability of the base triple 135, 136. A recent study developed a time-resolved fluorescence resonance energy transfer (FRET)-based high-throughput screening platform and identified novel small-molecule ligands (kynuramine derivatives) that destabilize the MALAT1 RNA triple helix, providing a new strategy for discovering chemical probes targeting lncRNA triple helices 137. In addition, clinically used small-molecule drugs can dock directly with RNA triple helices, offering the potential to accelerate translational validation due to their established safety profiles, as exemplified by chlorhexidine and kanamycin; however, further cellular and *in vivo* testing remains necessary 138. In contrast, the MENβ triple helix has been less explored: compounds such as emodin, GW5074, and L-798106 bind to the triple helix but exhibit limited inhibitory or toxic effects, highlighting the need for additional screening or conjugation strategies 139. Reverse transcription quantitative polymerase chain reaction (RT-qPCR) can be used to assess the stability of RNA triple helices, whose Cycle threshold (Ct) is determined by the reverse transcriptase extension efficiency, allowing for the simultaneous detection of both stable and unstable ligands, thereby supporting bidirectional functional interrogation 140. Mousseau et al. recently conducted a systematic evaluation of eight triplex-targeting molecules, confirming that their primary binding site is the MALAT1 major-groove triple helix rather than adjacent duplex regions. Furthermore, different small molecules display varying sensitivities to triple-helical length, base composition, and A-minor pocket conformation. Flavonoids primarily recognize base composition and A-minor pocket conformation, whereas alkaloids and berenil show a stronger preference for recognizing triple-helical length. Moreover, with the exception of neomycin, most interactions with both precursor and mature MALAT1 conformations are rapid and reversible, suggesting their applicability in dynamic regulatory contexts 141. Table 2 lists detailed information on small molecules targeting RNA triple helices. Most currently identified triple-helix-targeting small molecules still require further structural refinement or affinity optimization to enhance their potency.

Emerging strategies that use small molecules as probes to recruit nucleases or degradation effectors, thereby selectively reducing harmful transcripts containing triple helices, are gradually attracting attention. These approaches mainly include RNA-targeting chimeras (RNATACs) (Figure 6B) and Proximity-Induced Nucleic Acid Degraders (PINADs), which are new molecular modalities based on the proximity-induced strategy 142. PINADs achieve catalytic cleavage by incorporating imidazole groups—which mimic the active site of RNase A—into RNA-targeting small molecules (rSMs) localized to triple helix regions or other specific three-dimensional structures, thereby selectively cleaving abnormally stable or pathogenic RNA triple helices 143-145. The design of RNATACs is inspired by the principle of Proteolysis Targeting Chimeras (PROTAC), but their targets are RNA rather than proteins. Their basic architecture is a heterobifunctional small molecule: one end binds to a specific RNA structure (e.g., a triple helix), while the other end recruits intracellular RNA effectors (e.g., RNase L, Regnase-1, or SMG6) to mediate degradation 146. Recently, researchers have reported PINAD-1, the first bifunctional small-molecule degrader targeting the 3′-terminal triple helices of MALAT1, which couples a triplex-binding ligand to an imidazole-based cleavage warhead to achieve proximity-induced and selective MALAT1 degradation, while demonstrating that efficient RNA degradation depends not only on proximity but also on local triplex geometry and binder-induced destabilization 147. Further optimization of the ligandability and tissue-specific expression of RNA effectors is still required, along with the development of additional usable rSMs.

#### Antiviral targets

The RNA triple helix within the ENE element of KSHV PAN RNA protects the transcript from host-mediated degradation, while PAN itself drives global lytic gene activation and contributes to evasion of innate immunity, making it essential for efficient lytic replication 119. Knockdown of the PAN gene at its source suppresses nuclear mRNA export and reduces cytoplasmic accumulation of transcripts encoding late lytic viral proteins 148. However, deletion of ENE does not directly impair lytic replication but instead increases cytoplasmic RNA levels 47, indicating that direct engineering of the ENE triple helix is not a viable approach. Therefore, efforts should focus on exploring small molecules that target ENE to directly or indirectly modulate the activity of functional RNA, with the potential to obtain KSHV-specific antiviral agents; this strategy may also be applicable to other viral infections involving RNA triple helices or similar structures containing ENEs.

Using the SMM screening strategy, compounds capable of specifically recognizing and binding to RNA triple helices were identified and subjected to biochemical analyses. Ultimately, a small-molecule ligand was identified that specifically binds to both the PAN triple helix and the pocket formed by the AU dinucleotide bulge at its base (Figure 4A) 124. This compound is likely to disrupt the interaction between the triple helix and the minor capsid protein ORF26, thereby impacting KSHV pathogenesis and presenting a new therapeutic strategy that targets a viral lncRNA instead of a host factor 124. Notably, such single-stranded RNA bulges forming pocket-like structures are present in many ENE-containing triple helices 14, and they may represent important sites for future small-molecule targeting. These results indicate that the pocket-like bulges at the base of ENE-containing triple helices constitute structurally privileged small-molecule-druggable hotspots, where ligands identified via SMM screening can selectively perturb triplex-protein interactions and potentially destabilize pathogenic viral RNAs such as PAN.

#### Oligonucleotide therapy

Antisense oligonucleotides (ASOs) are short (typically 12-30 nt) synthetic oligonucleotides that bind to target RNAs (and occasionally DNAs) through Watson-Crick or Hoogsteen base pairing, thereby regulating the processing, stability, or translation of nucleic acids, which in turn alters gene expression or exerts therapeutic effects 149. ASOs can disrupt RNA's secondary and tertiary structures via competitive binding or by inducing conformational changes, making transcripts more prone to degradation or functional loss 150, 151. Thus, ASOs can be designed to disrupt or block the formation and stabilization of pathogenic RNA triple helices (Figure 6C). When ASOs are directed to the U-rich main strand or the A-rich complementary strand regions required for triplex formation, they can competitively replace the natural third strand or prevent closure of the triple helix 110. In MALAT1, ASO-mediated disruption of the triple helix exposes the 3′ end, abolishes ENE protection, and promotes rapid exonucleolytic degradation. Competitive displacement assays further showed that locked nucleic acid (LNA) 15 and its phosphorothioate-modified derivative—phosphorothioate LNA 15 (PS-L15)—can displace A-tract and the RNA triple-helix-binding protein METTL16 from the triple helix, forming an RNA·LNA-RNA triple helix, and markedly reduce MALAT1 and MENβ expression 152, representing a practical dual-target inhibition strategy. These studies establish ASOs (particularly chemically optimized, triplex-targeting designs such as LNA and PS-LNA oligonucleotides) as versatile tools for selectively dismantling pathogenic RNA triple helices, while simultaneously destabilizing oncogenic lncRNAs (such as MALAT1/MENβ) and eliminating toxic repeat-expanded transcripts in neurodegeneration, thereby offering a broadly applicable therapeutic strategy across diverse RNA structure-driven diseases.

In addition, bifacial peptide nucleic acids (bPNAs), which are built on a peptide scaffold attached with bases every two amino acids, can be designed to target RNA triple helices and induce structural displacement, thereby modulating the activity of the parent RNA. This approach exploits the ability of the artificial base melamine to form an UMU base triple with two uridines in the URIL of an RNA triplex 153-155 and competitively displace the natural poly(A) tail 128, 156-158. Since the melamine base in bPNAs can recognize uridine-rich RNA motifs, this strategy may be applicable to a variety of RNA targets. On this basis, several bPNA scaffolds, including normal peptide, diketopiperazine, and isodipeptide backbones, have been developed to target the MALAT1 triple helix. Among these, the K2M-A-K2M tripeptide (a tripeptide-based bPNA containing four melamine groups, hence also termed 4M bPNA) exhibits the best activity, reducing MALAT1 RNA half-life to 0.5 h and suppressing MALAT1 expression by approximately 50% in PANC-1 pancreatic cancer cells 159. This strategy provides a new avenue for lncRNA-targeted cancer therapy, particularly for oncogenic transcripts whose stability depends on a triple helix.

## Challenges and Strategies

### Improving target specificity and avoiding off-target effects

RNA triple helices often contain conserved or repeated nucleoside patterns and interact with other RNAs or proteins within the cell; small molecules or oligonucleotides tend to bind non-specifically to similar sequences or structures, leading to off-target effects 48. To overcome the low selectivity caused by sequence or structural similarity, a structural recognition approach can be considered instead of relying solely on sequence recognition. This involves utilizing high-resolution structural information to locate specific triple interfaces or peripheral anchoring secondary structures. In drug design, one can consider introducing ligand features capable of recognizing specific sites on triple helices (e.g., ligands that recognize the U·A-U geometric feature unique to triples) 107. These high-quality structural and dynamic data can significantly enhance the efficiency of selective screening. To address potential hybridization issues between oligonucleotides and non-target transcripts, ASOs can undergo chemical modification and length optimization to improve affinity and reduce non-specific binding. Additionally, *in vivo* off-target prediction at the sequence level and RNA sequencing (RNA-seq) can be employed to screen candidates 149, 160. For the screening of highly specific small-molecule ligands, a multimodal screening workflow (high-throughput binding screening → structural validation → intracellular functional validation) can be employed, incorporating bispecific molecules to enhance functional selectivity and direct therapeutic efficacy 142, 161, 162. Additionally, telomerase and spliceosomal RNA triple helices represent mechanistically compelling but still underexploited therapeutic targets. Although current telomerase-directed and spliceosome-modulating agents mainly act on the hTR template region, telomerase-associated pathways, or spliceosomal protein components rather than the triple helices themselves, these systems provide important precedents for future structure-guided intervention against disease-relevant RNA triplex motifs 163, 164.

### Overcoming the dynamics and complexity of RNA structure

The RNA triple helix is not a rigid structure; its folding, formation and disassembly are influenced by post-transcriptional processing, protein binding, modifications, and the ionic environment, leading to variations in the accessibility of target sites under different conditions. If kinetic factors are not taken into account, designed ligands may be ineffective *in vivo* 110. Before the RNA triple helix is fully processed, ligand molecules or ASOs can be designed to bind to its folded intermediate states, thereby preventing the triple helix from fully forming and stabilizing, which relies heavily on relevant structural and kinetic data 165. Establishing drug-binding sites or serving as co-targeting sites through chemical modification is another strategy 55, which can ignore the dynamics of RNA structure to the greatest extent possible. In addition, given that many RNA triple helices and their corresponding RBPs constitute functional units, it is possible to target the interacting RBPs, such as by connecting small molecules to protein degradation modules 142, thereby bypassing the RNA itself and directly intervening in the overall function of the triple helix. Although RNA triple-helix-based drug treatment strategies are diverse, they still face many challenges and require refinement in numerous aspects before they can ultimately be applied clinically to benefit mankind. Moreover, since many RNA triple helices reside within the cell nucleus, current delivery systems face challenges in efficiently and selectively penetrating the nucleus, which further limits the development of therapeutics against these targets 166-168. Finally, RNA secondary structures play a crucial role in regulating antigen expression and the immunogenic efficacy of mRNA vaccines by modulating translation initiation, transcript stability, and innate immune sensing 36, 37. Given the reported roles of RNA triple helices in inhibiting RNA decay 16 and promoting both transcription 14 and translation 48, these structures may offer a promising avenue for enhancing RNA stability and translational efficiency in mRNA-based therapeutics. Rosemann et al. showed that *Malat1*-derived 3′-end motifs, including the triple-helix element, could support translation of synthetic reporter mRNAs in human cells, although they consistently underperformed canonical poly(A) tails, suggesting that triple helices may serve as partial poly(A)-replacement elements but still require further optimization 56.

## Conclusions

As an important motif in higher-order nucleic acid structures, research on RNA triple helix is gradually shifting from basic structural studies to interdisciplinary frontier areas such as functional dissection and disease therapy. Over the past several decades, researchers have progressed from the initial discovery of RNA triple helices in artificial systems to the continuous identification of natural examples in viral, prokaryotic, and eukaryotic RNAs. This has gradually revealed the central role of RNA triple helices in key biological processes, including RNA stabilization, transcriptional and translational regulation, maintenance of telomerase activity, ligand recognition and regulation mediated by riboswitches, control of transposition, and RNA splicing. With the rapid development of structural biology and high-throughput techniques, such as cryo-EM, single-molecule FRET, and selective 2′-hydroxyl acylation analyzed by primer extension and mutational profiling (SHAPE-MaP), our understanding of the structural diversity, dynamic equilibria, and biological significance of RNA triple helices has continued to deepen.

At disease level, accumulating evidence indicates that the formation, disruption, or aberrant stabilization of RNA triple helices is closely associated with a variety of pathological processes, including cancer, viral infections, neurodegenerative disorders, and genetic diseases. Therapeutic strategies targeting these structural abnormalities are gradually shifting from functional validation to targeted intervention. Also, small-molecule ligands, antisense oligonucleotides, siRNAs, CRISPR, and emerging RNA degradation technologies are opening new directions for structured RNA-targeting drugs. This marks the transition of RNA triple helices from a concept in structural biology to a potential druggable structural motif.

Overall, RNA triple helices are essential and highly diverse functional elements within gene regulatory networks. Positioned at the intersection of RNA structure and function, they directly encode structural information into RNA folding through processes such as stability regulation, catalytic activity modulation, ligand recognition, and protein recruitment, thereby bridging the gap between basic RNA biology and clinical applications.

## Supplementary Material

Supplementary figures and tables.

## Figures and Tables

**Table 1:** Information on the resolved triple-helix structures (ordered numerically and alphabetically).

Structure	Method and resolution	PDB ID	References
P2b-P3 pseudoknot from human telomerase RNA	Solution NMR	1YMO	8
P2B-P3 pseudoknot of human telomerase RNA	Solution NMR	2K95, 2K96	22
Pyrimidine motif triple helix in the *Kluyveromyces lactis* telomerase RNA pseudoknot	Solution NMR	2M8K	82
PreQ1 Class II riboswitch from *Streptococcus pneumoniae*	Solution NMR	2MIY	67
SAM-II riboswitch bound to S-adenosylmethionine	X-ray diffraction, 2.80 Å	2QWY	31
Self-spliced group II intron	X-ray diffraction, 3.10 Å	3BWP	10
Yeast spliceosome	Electron microscopy, 3.60 Å	3JB9	169
ENE, a viral RNA stability element, in complex with A9 RNA	X-ray diffraction, 2.50 Å	3P22	11
C-di-GMP-II riboswitch from *Clostridium acetobutylicum* bound to c-di-GMP	X-ray diffraction, 2.51 Å	3Q3Z	170
Class II preQ1 riboswitch	X-ray diffraction, 2.28 Å	4JF2	66
Triple-helical stability element at the 3**′** end of MALAT1	X-ray diffraction, 3.10 Å	4PLX	13
PreQ1 Riboswitch	X-ray diffraction, 2.75 Å	4RZD	62
Group II intron complexed with its reverse transcriptase	Electron microscopy, 4.50 Å	5G2Y	171
Catalytic Step I spliceosome	Electron microscopy, 3.40 Å	5GMK	102
*Tetrahymena* telomerase RNA pseudoknot	Solution NMR	5KMZ	172
Human spliceosome activated for step 2 of splicing	Electron microscopy, 5.90 Å	5MQF	28
*Thermobifida fusca* guanidine III riboswitch with guanidine	X-ray diffraction, 1.91 Å	5NWQ	63
Group II intron lariat with an intact 3**′** splice site (pre-2s state)	X-ray diffraction, 3.70 Å	6CHR	94
MetY SAM V riboswitch	X-ray diffraction, 2.50 Å	6FZ0	64
Structure of a group II intron retroelement after DNA integration	Electron microscopy, 3.60 Å	6MEC	95
U**·**A-U-rich RNA triple helix with 11 consecutive base triples	X-ray diffraction, 2.50 Å	6SVS	20
Stabilized PAN ENE bimolecular triplex with a GC-clamped polyA tail, in complex with Fab-BL-3,6	X-ray diffraction, 3.30 Å	6X5N	124
Double-ENE RNA stability element in complex with a 28-mer poly(A) RNA	X-ray diffraction, 2.89 Å	7JNH	16
B dENE construct complexed with a 28-mer poly(A)	Electron microscopy, 5.60 Å	7LJY	46
NAD^+^-II riboswitch	X-ray diffraction, 2.15 Å/2.50 Å	8GXB, 8GXC	65
NAD-II riboswitch (two strands)	X-ray diffraction, 1.67 Å	8I3Z	30
Ribosomal large subunit from* Entamoeba histolytica*	Electron microscopy, 2.80 Å	9V1I	17

**Table 2:** Summary of RNA triple-helix-targeting small molecules (chemical structures, shown in the Figure S1, are ordered alphabetically).

Small molecule	Targeted triple helix	Effect on triple helix	References
Aromatic heterocyclic compound M5	MALAT1 triple helix	Disrupting the stability of triple helix	135, 136
Benzoquinoquinoxaline-neomycin conjugate	U·A-U RNA triple helix	Enhancing the thermal stability of triple helix	173
Berberine, coralyne, palmatine	U·A-U RNA triple helix	Enhancing the thermal stability of triple helix	40
Berenil (diminazene aceturate)	U·A-U RNA triple helix	Promoting the thermal stability of triple helix	7
Chelerythrine	U·A-U RNA triple helix	Increasing binding affinity and the thermal stability	174
Chlorhexidine, kanamycin	MALAT1 triple helix	Predicted to bind to triple helix	138
Compound 15	PAN triple helix and two nucleotides bulge at the bottom	Enhancing resistance to nucleic acid exoribonucleases	124
Dibenzofuran and its derivatives	MALAT1 triple helix	Selective binding, stabilizing the structure and inhibiting degradation	15, 175
Diminazene derivatives	MALAT1 triple helix	Bidirectional regulation: Some reduce nuclease accessibility; others promote degradation	24
Emodin, GW5074, L-798106	MENβ/NEAT1_2 triple helix or immature fragments	Inhibitory effect decreases progressively	139
Fisetin	U·A-U RNA triple helix	Enhancing the thermal stability of triple helix	176
Imidazole compounds 5, 16	MALAT1 triple helix	Specific binding, MALAT1 RNA decreased in breast tumor models	133
Kynuramine class of derivatives compounds 7, 10, 15	MALAT1 triple helix	Destabilizing the triple helix through non-intercalative interactions	137
Luteolin	U·A-U RNA triple helix	Enhancing the thermal stability of RNA triple helix	141, 177
Methylene blue	U·A-U RNA triple helix	Stabilizing Watson-Crick strand, destabilizing Hoogsteen strand	41
Neomycin, gentamicin, paromomycin	U·A-U RNA triple helix	Enhancing the thermal stability of triple helix with neomycin showing the best	141, 178
N-fused quinazolino-quinazoline-dione 2z	MALAT1 triple helix	Recognizing triple helix UUG pockets; exhibiting toxicity to tumor cells with high expression of MALAT1	179
Phenosafranine	U·A-U RNA triple helix	Increasing binding affinity and the thermal stability	180
PINAD-1	MALAT1 triple helix	Binder-induced triplex destabilization	147
Quercetin	MALAT1 triple helix	Inducing structural changes, resulting in decrease in MALAT1 expression	134
[Ru(bpy)₂(uip)]²^+^ (Ru1) and [Ru(phen)₂(uip)]²^+^ (Ru2)	U·A-U RNA triple helix	Enhancing the thermal stability of RNA triple helix	181
[Ru(dmb)₂(dppz-idzo)]²^+^	U·A-U RNA triple helix	Enhancing the thermal stability of RNA triple helix	42
Sanguinarine	U·A-U RNA triple helix	Enhancing the thermal stability of triple helix	141, 182
Two types 9-amino alkyl berberine analogs BC1, BC2	U·A-U RNA triple helix	Increasing binding affinity, improving triple helix stability	183
