# Supplementary material for: RNA Triple Helices: From Structures and Mechanisms to Therapeutic Targets
Source: Int J Biol Sci. 2026 Aug 21;22(13):7447–70. doi: 10.7150/ijbs.132736 (PMC13541005; doi:10.7150/ijbs.132736)
Supplement: Supplementary file 1 [file ijbsv22p7447s1.pdf]

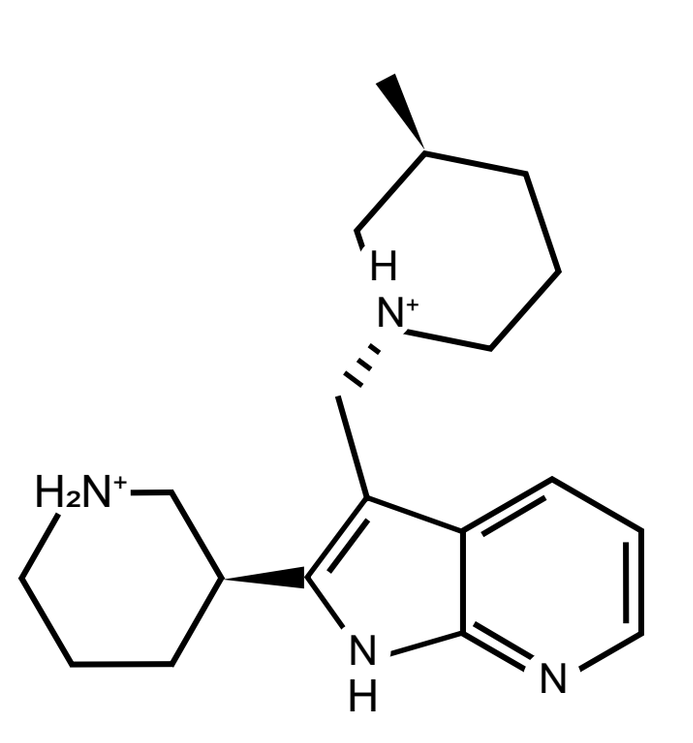

Aromatic heterocyclic compound M5

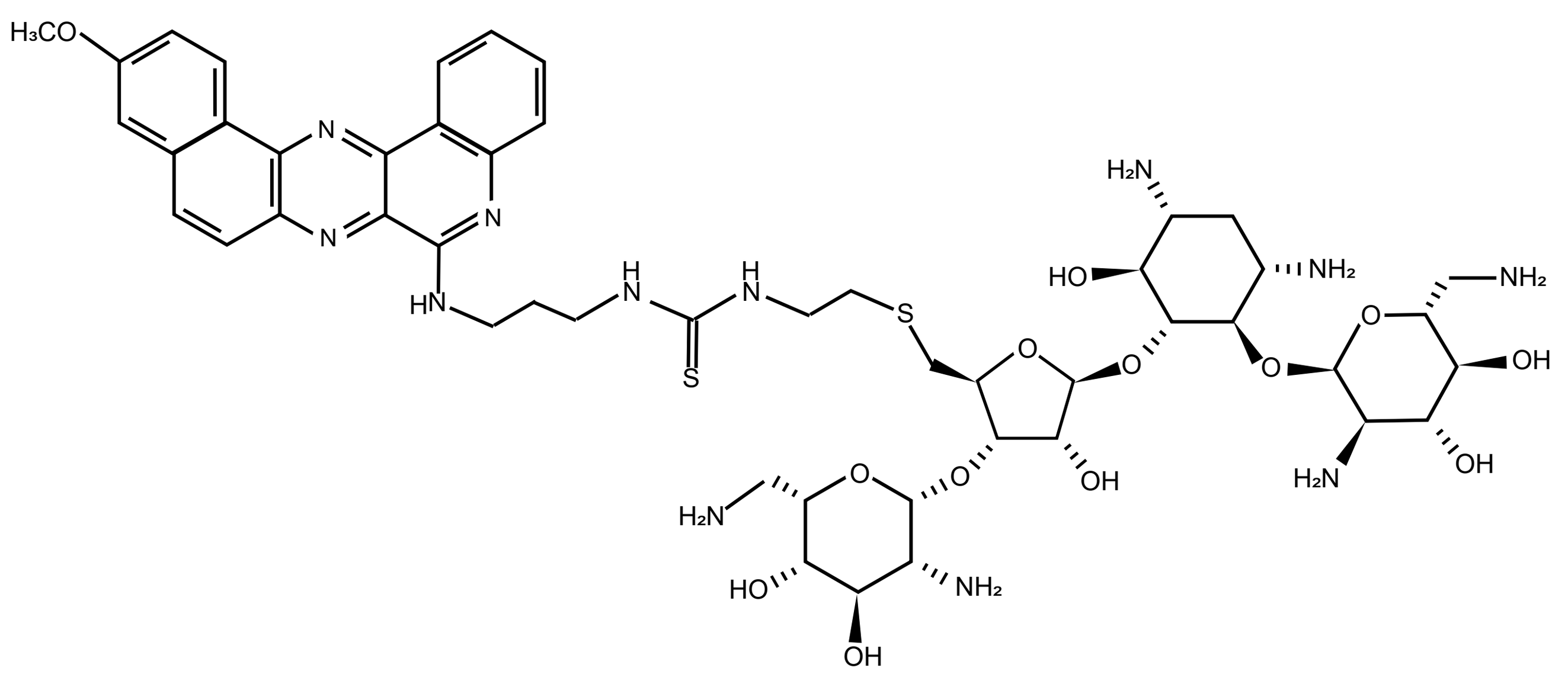

Benzoquinoquinoxaline-neomycin conjugate

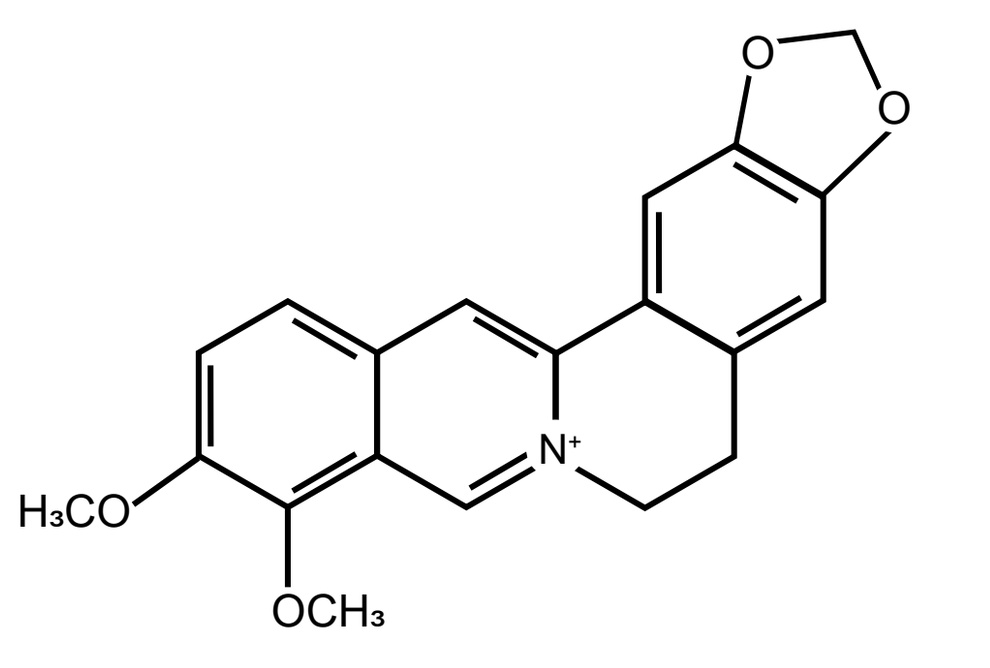

Berberine

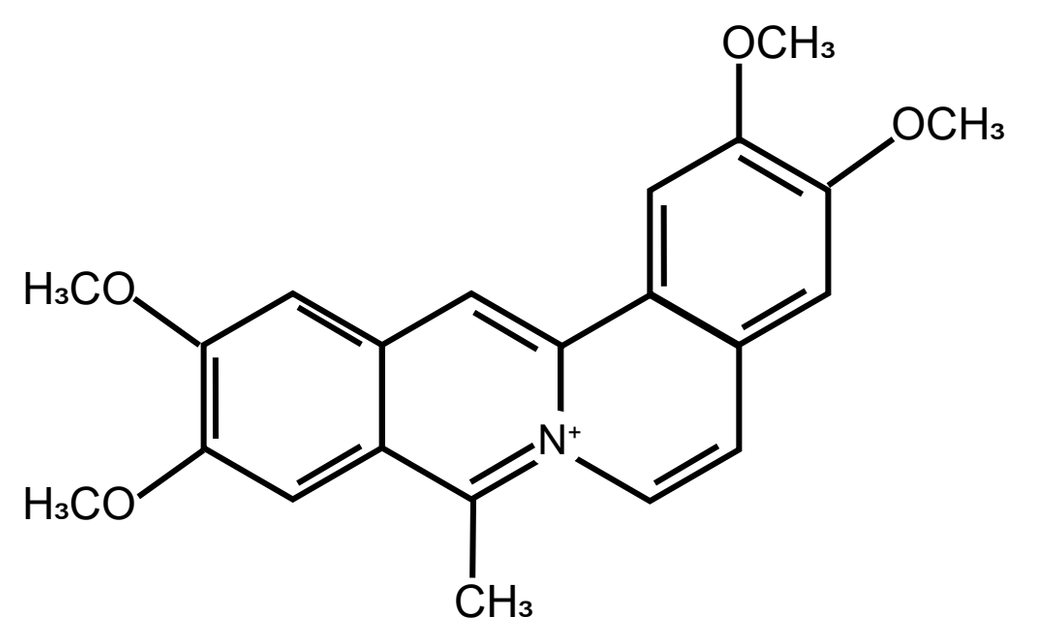

Coralyne

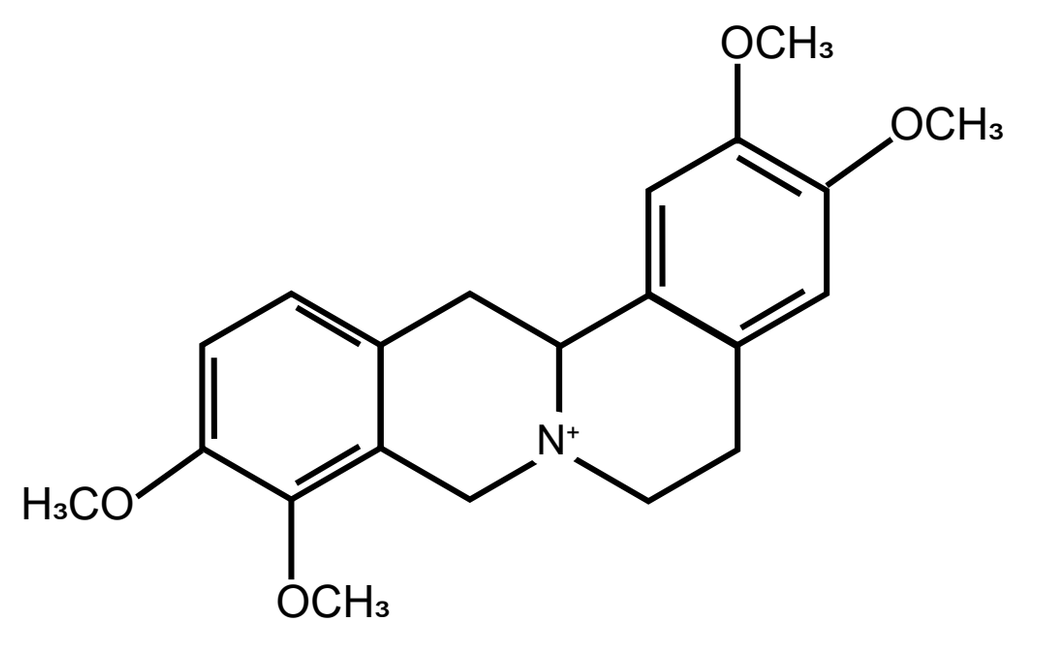

Palmatine

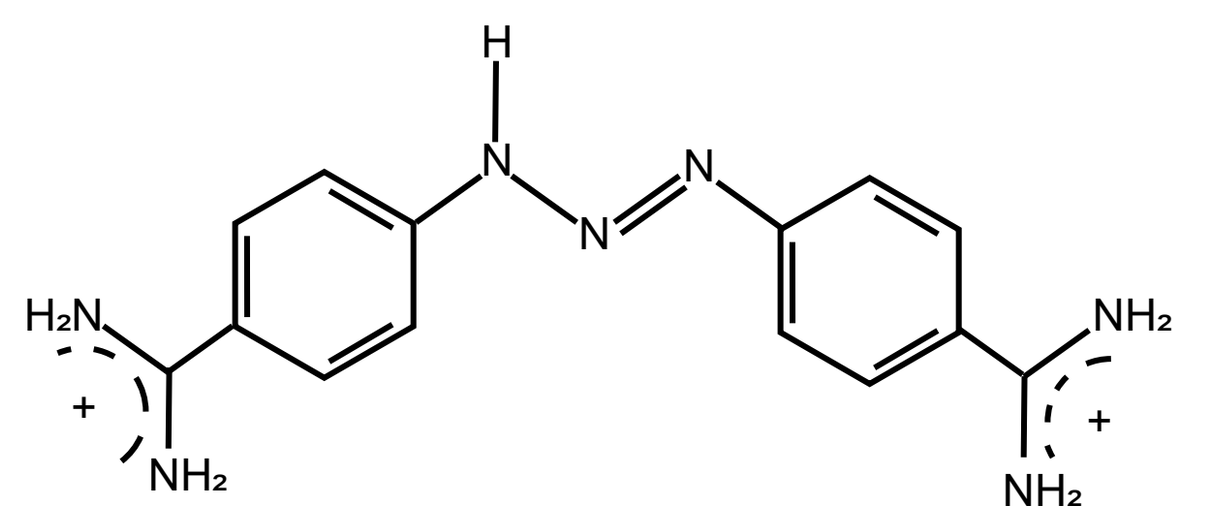

Berenil

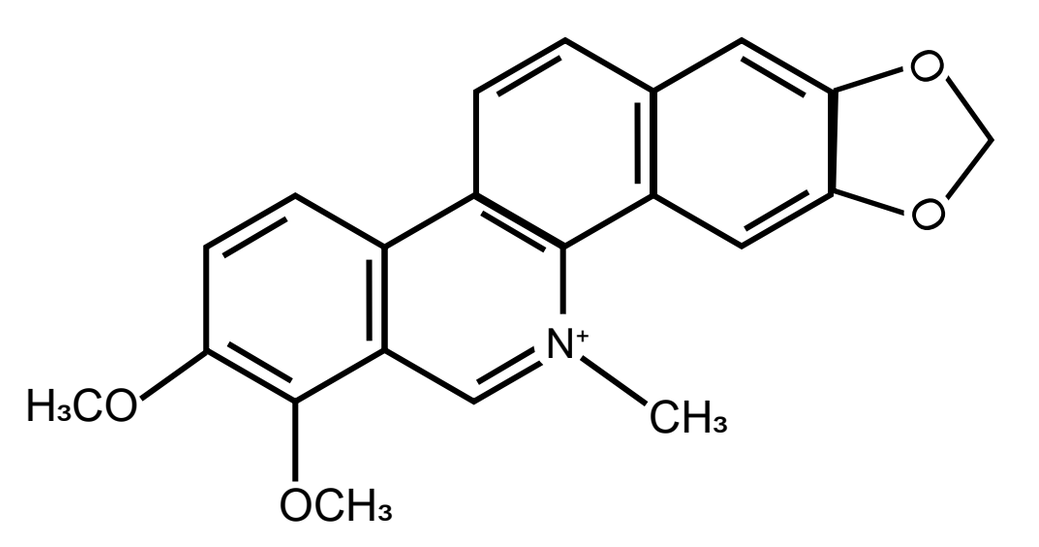

Chelerythrine

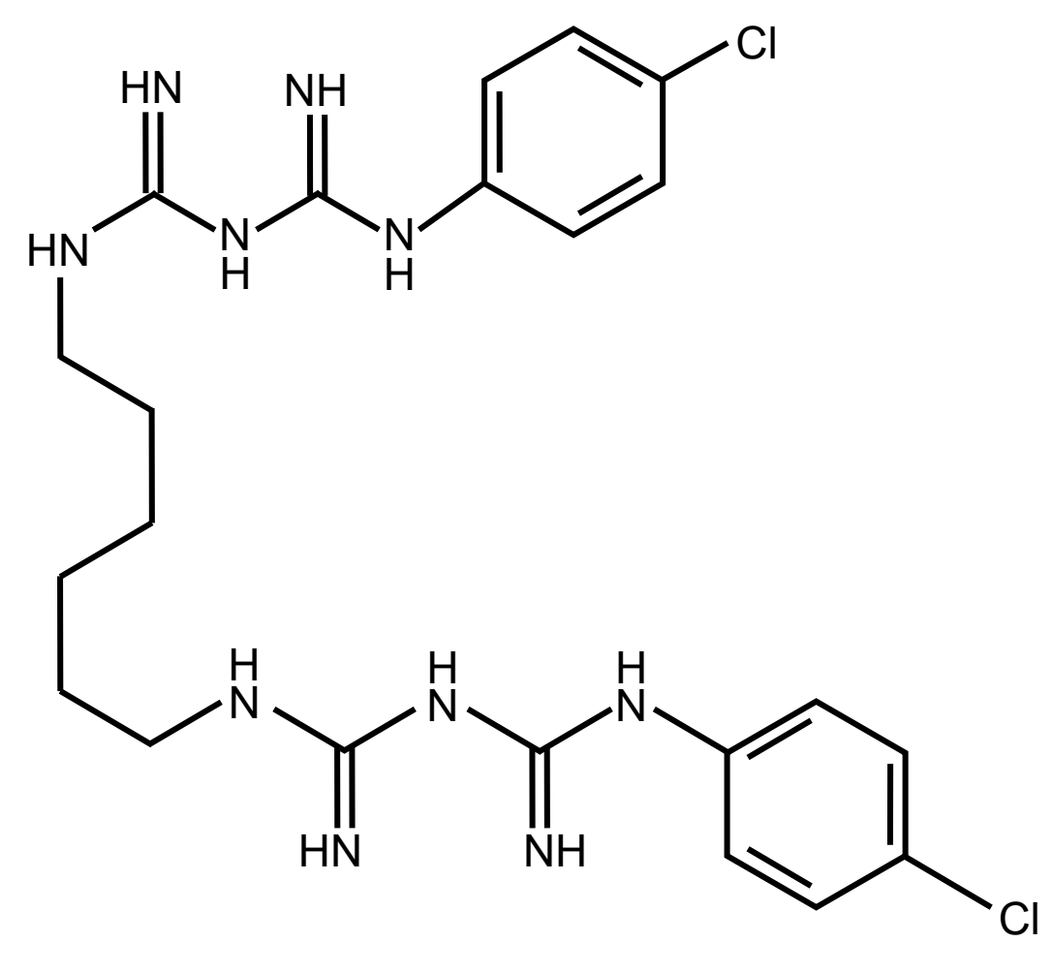

Chlorhexidine

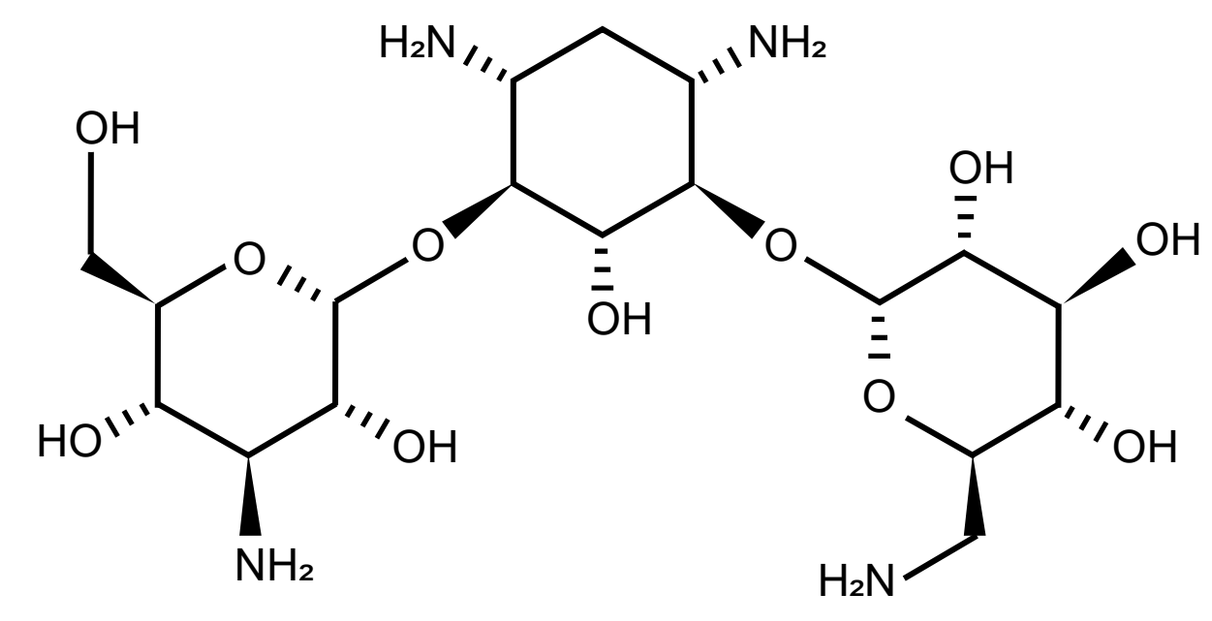

Kanamycin

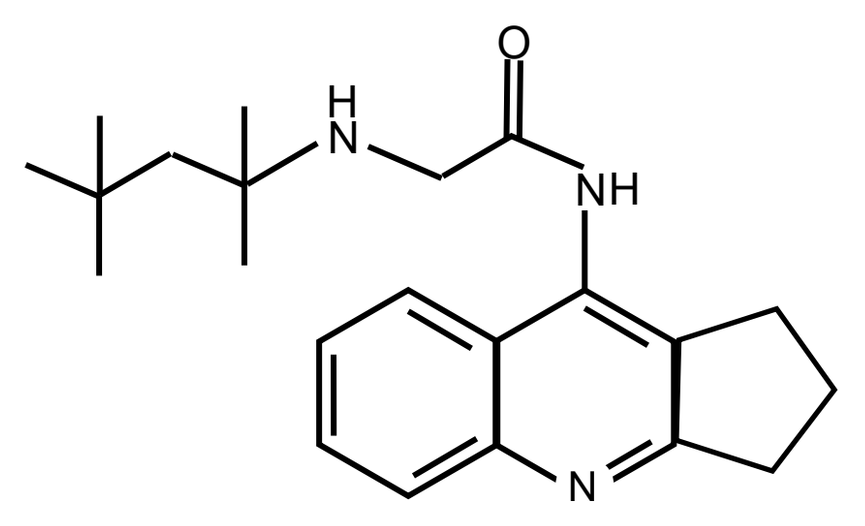

Compound 15

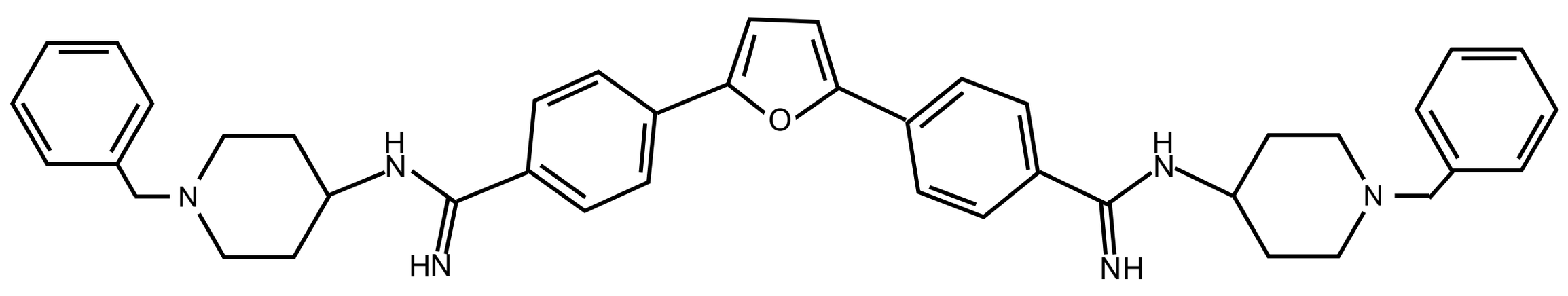

Diphenylfuran p8

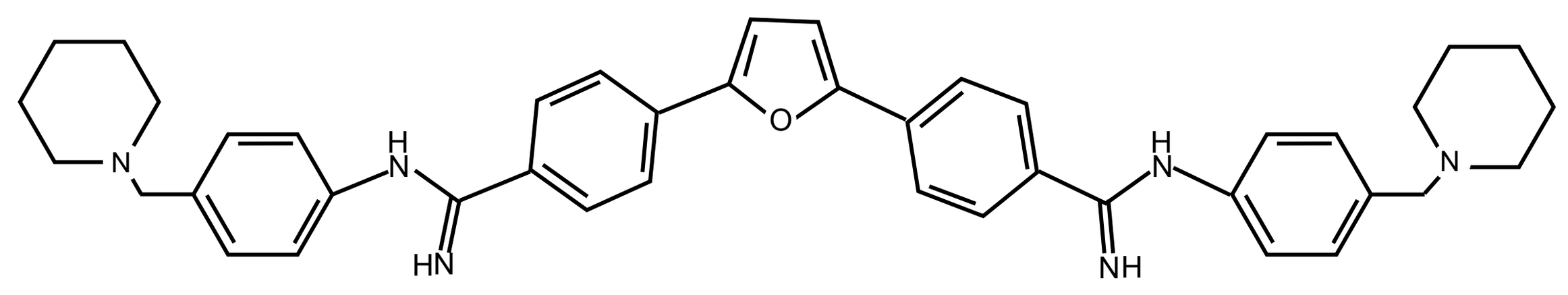

Diphenylfuran p20

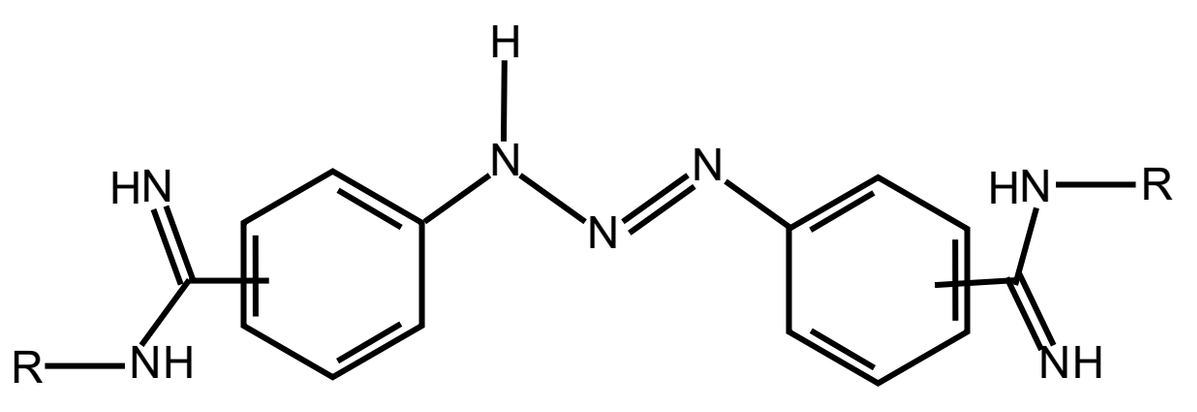

Diminazene derivatives

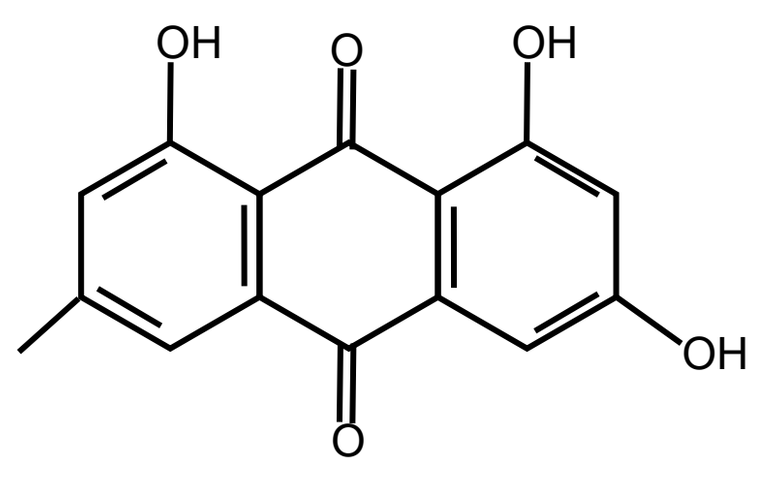

Emodin

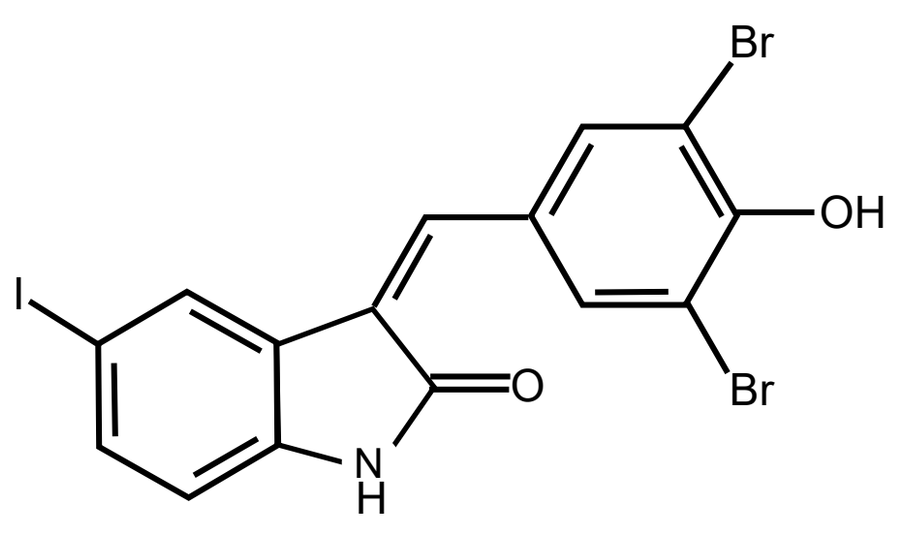

GW5074

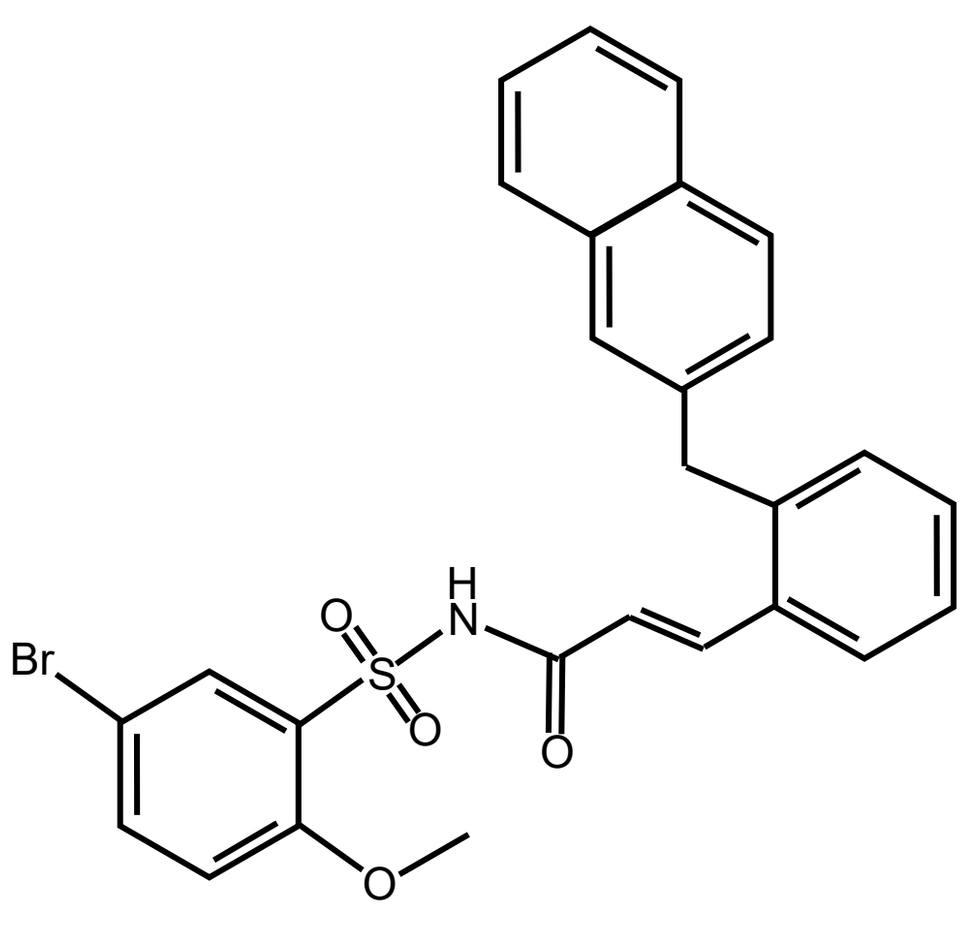

L-798106

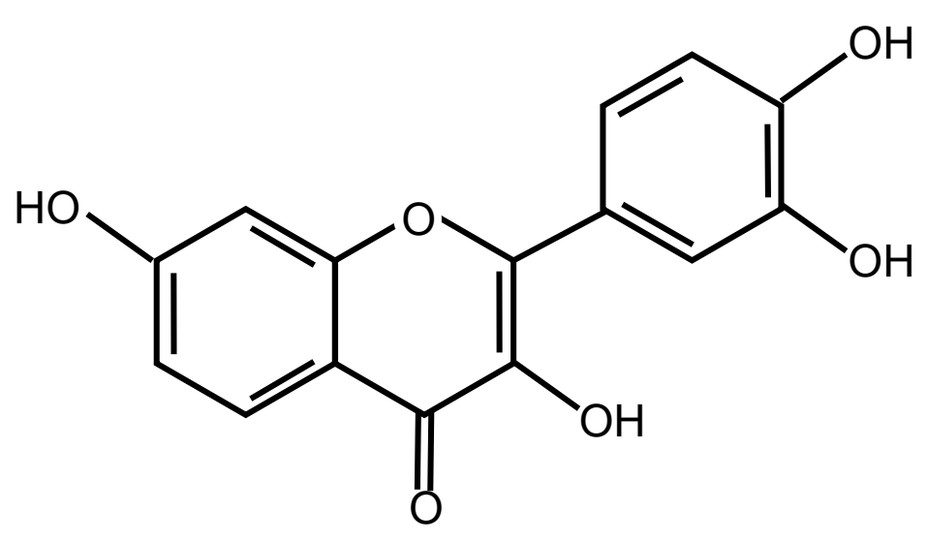

Fisetin

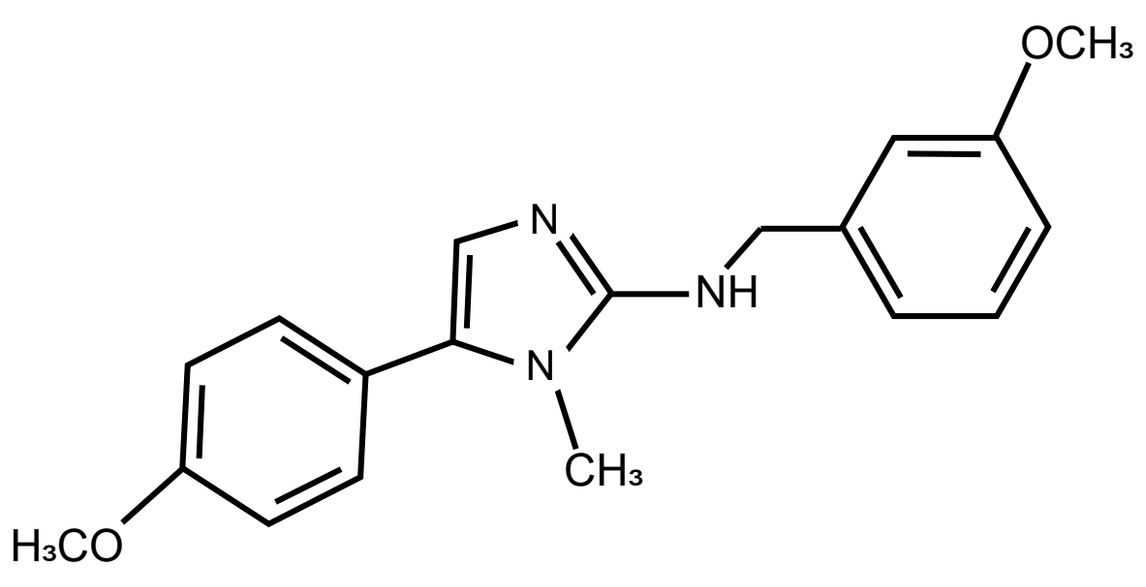

Imidazole compounds 5

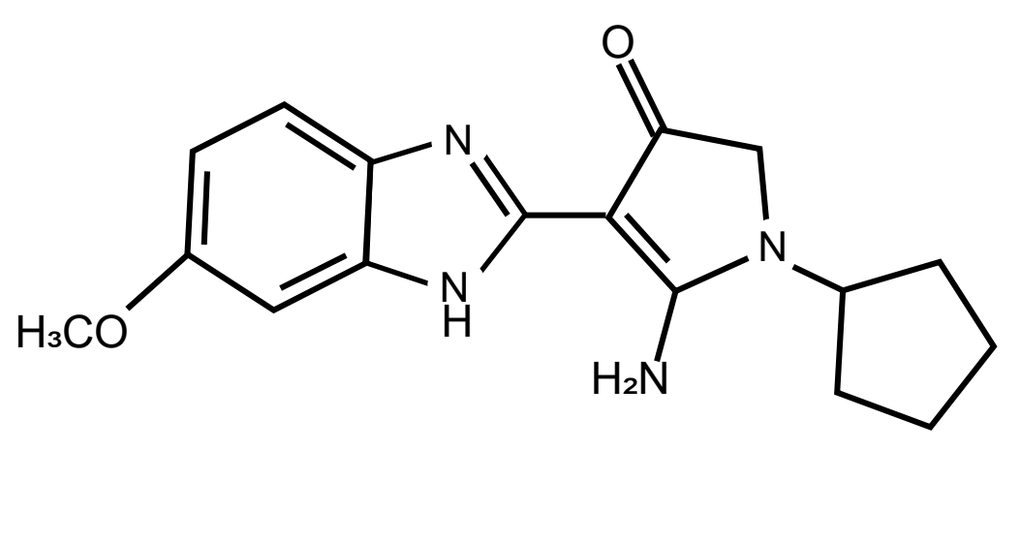

Imidazole compounds 16

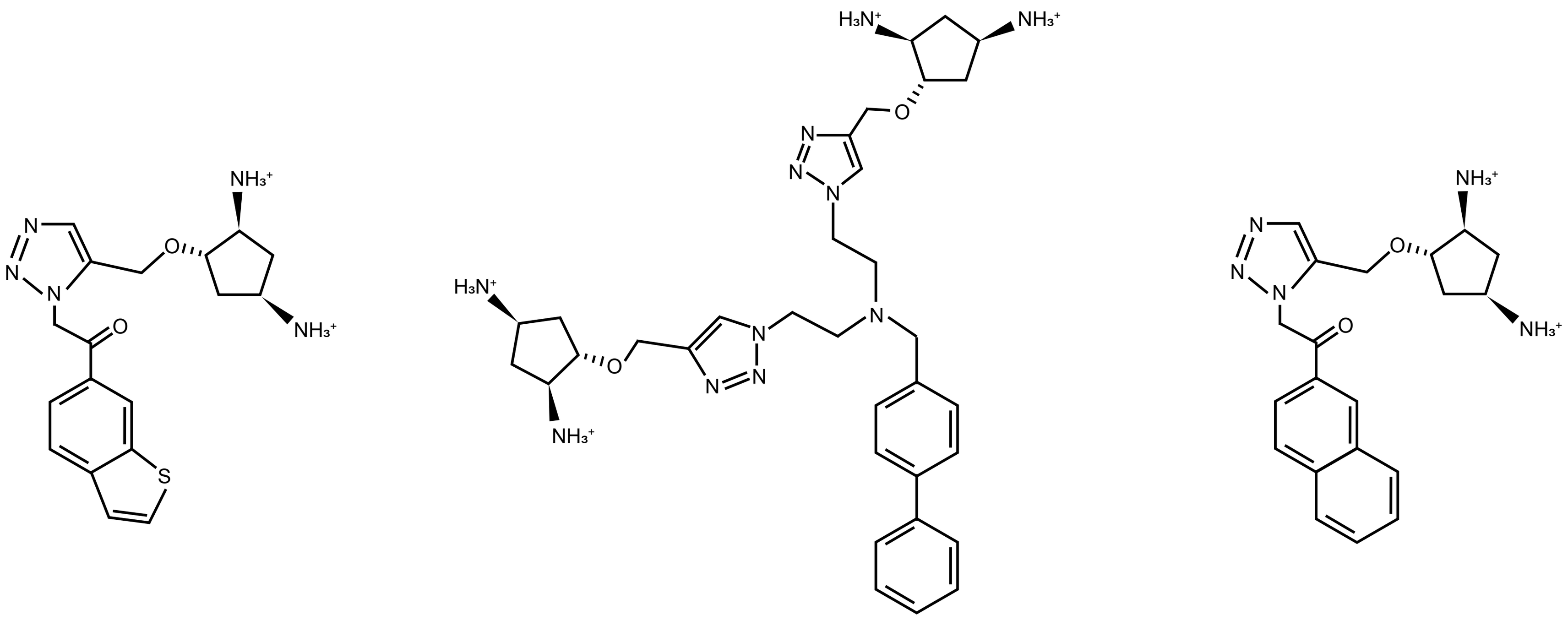

Kynuramine class of derivatives compounds 7, 10, 15

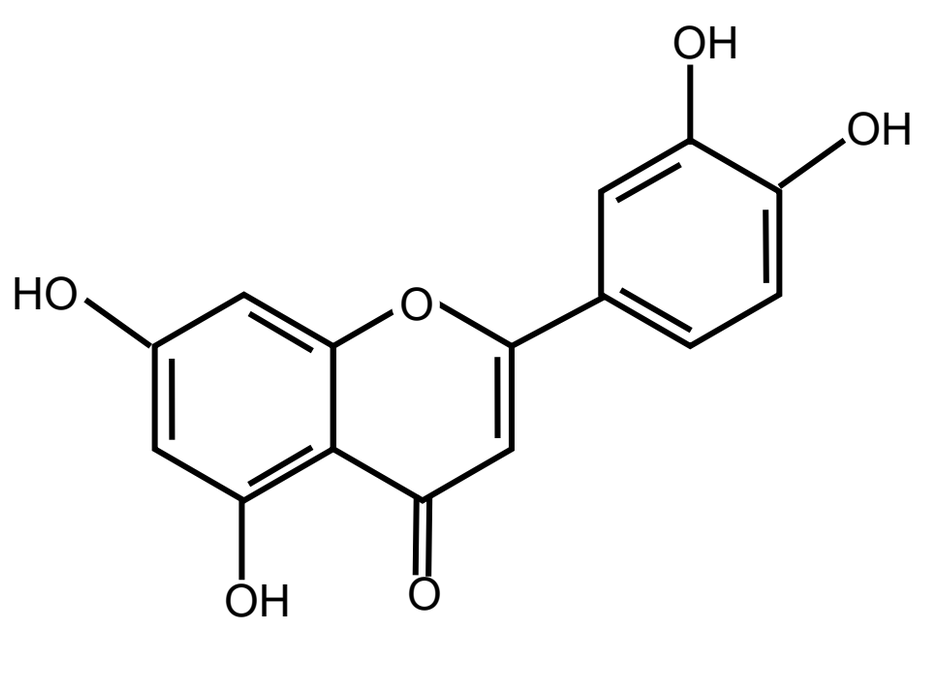

Luteolin

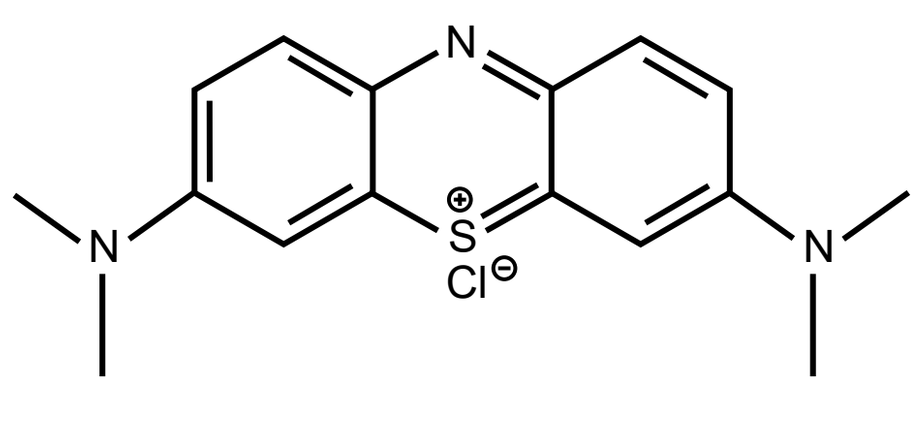

Methylene blue

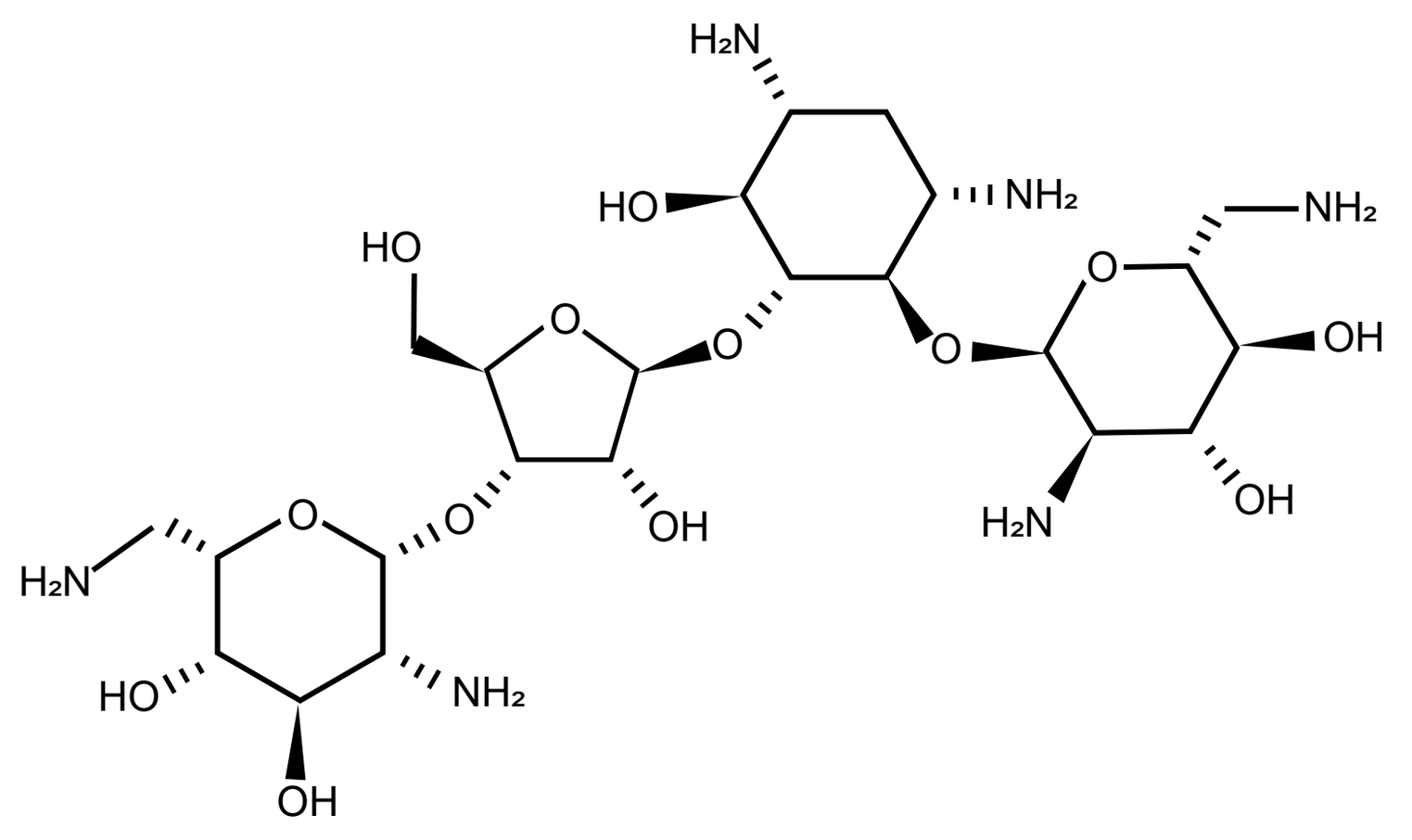

Neomycin

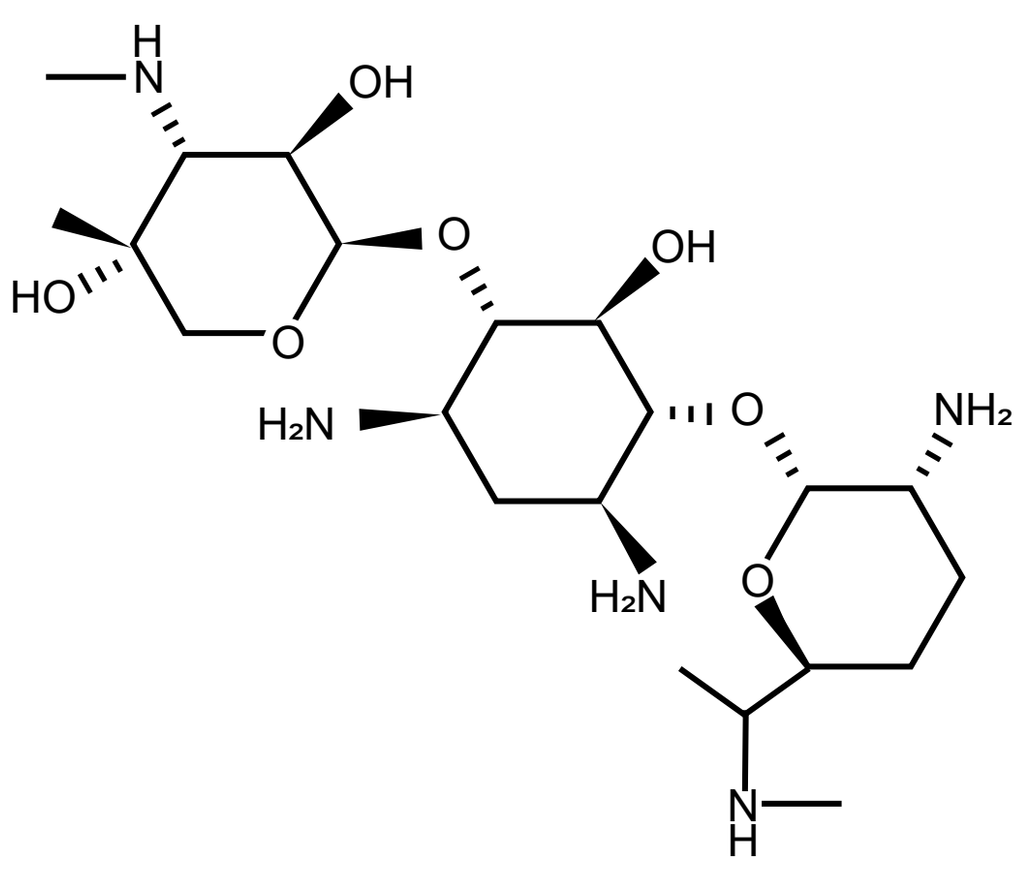

Gentamicin

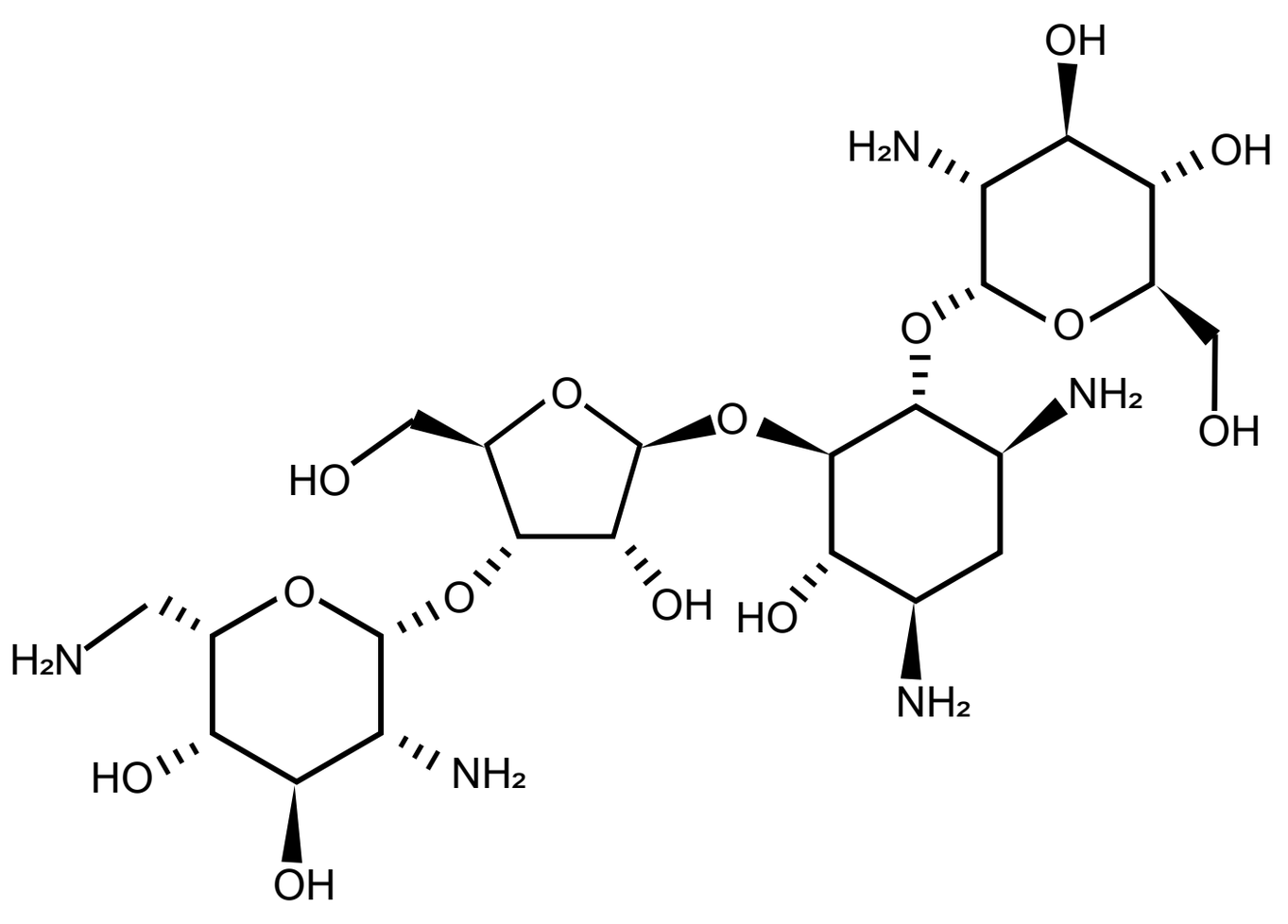

Paromomycin

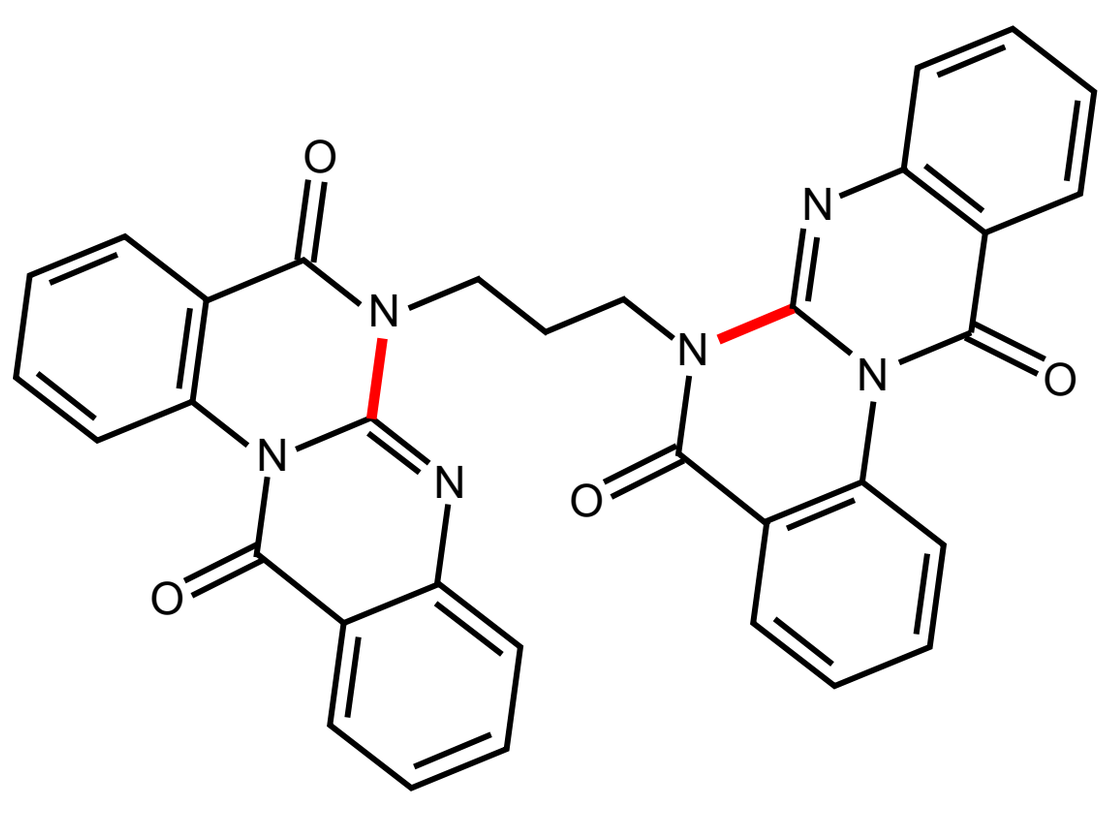

N-fused quinazolino-quinazoline-dione 2z

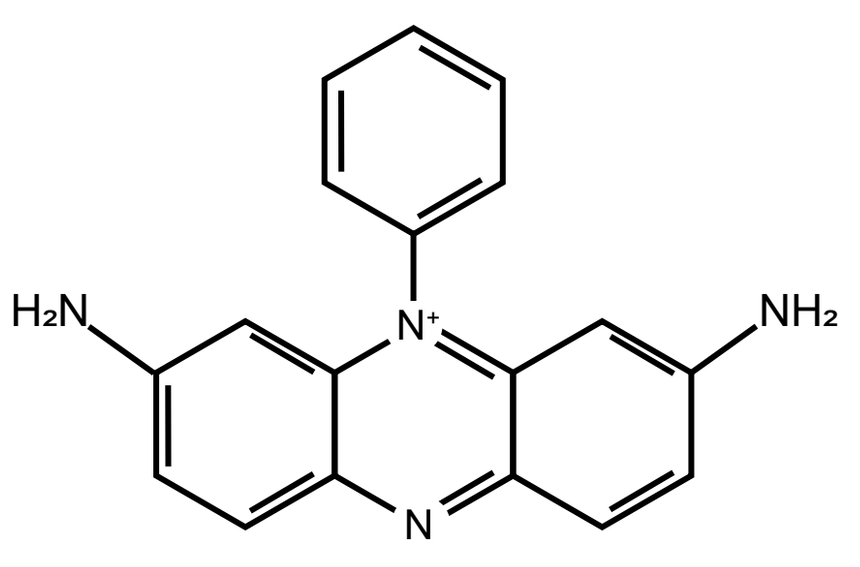

Phenosafranine

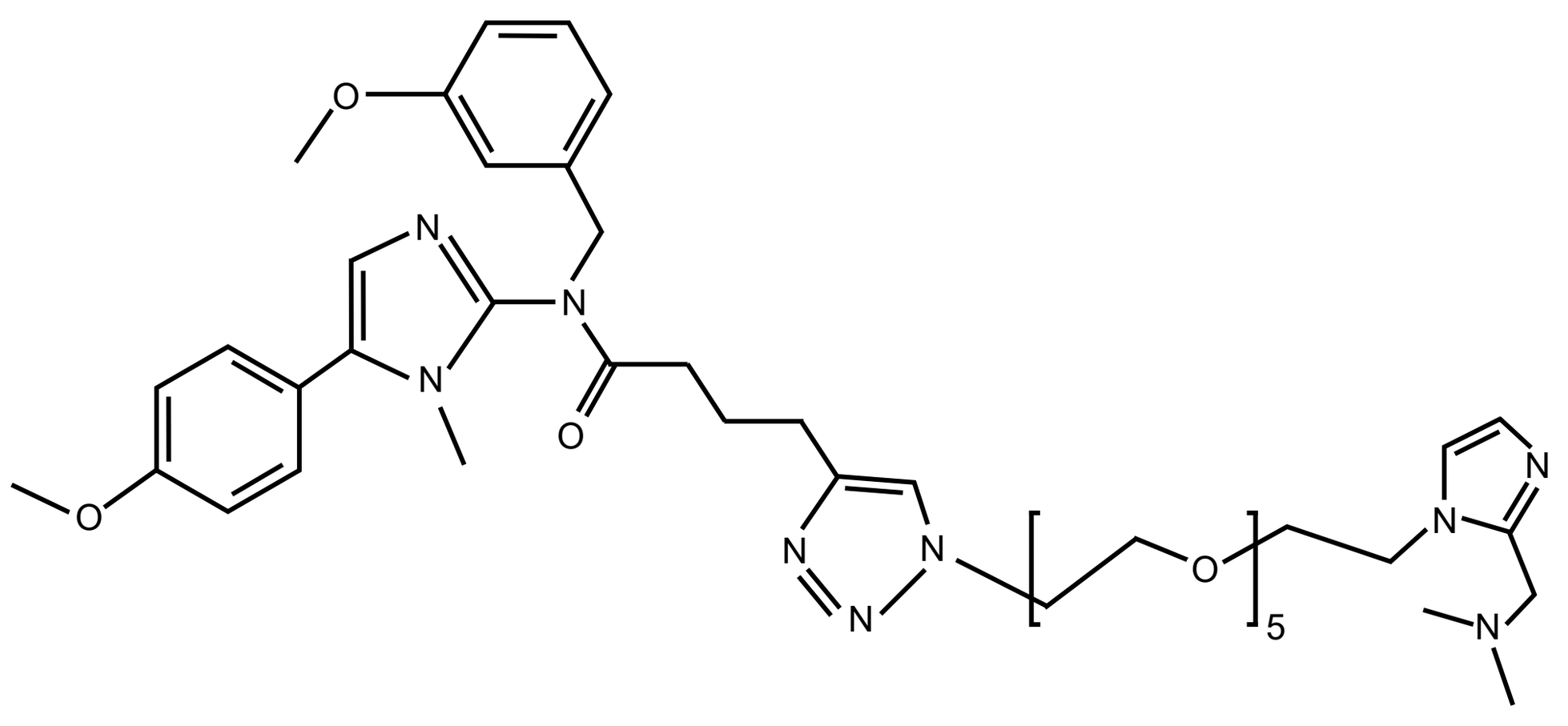

PINAD-1

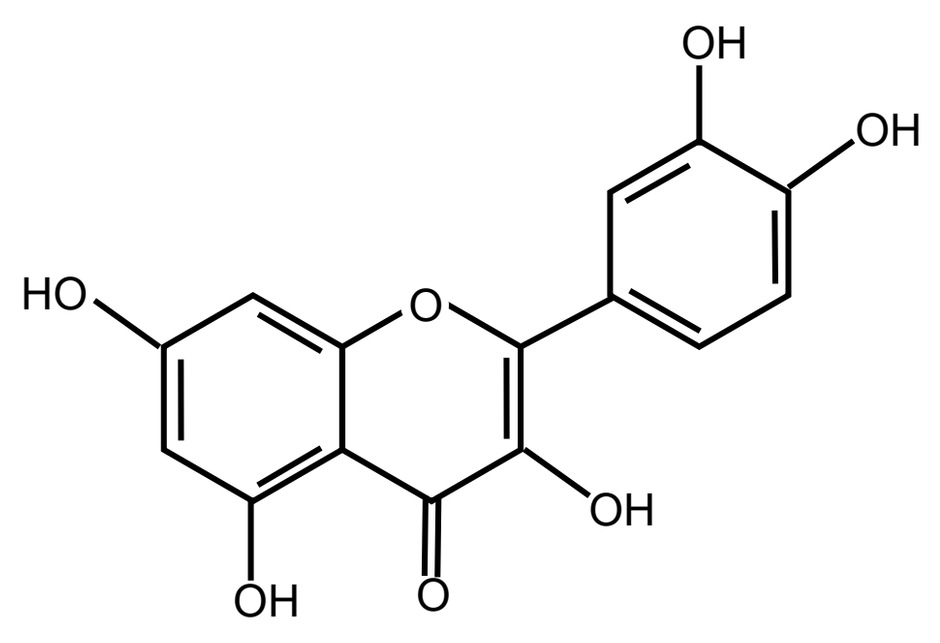

Quercetin

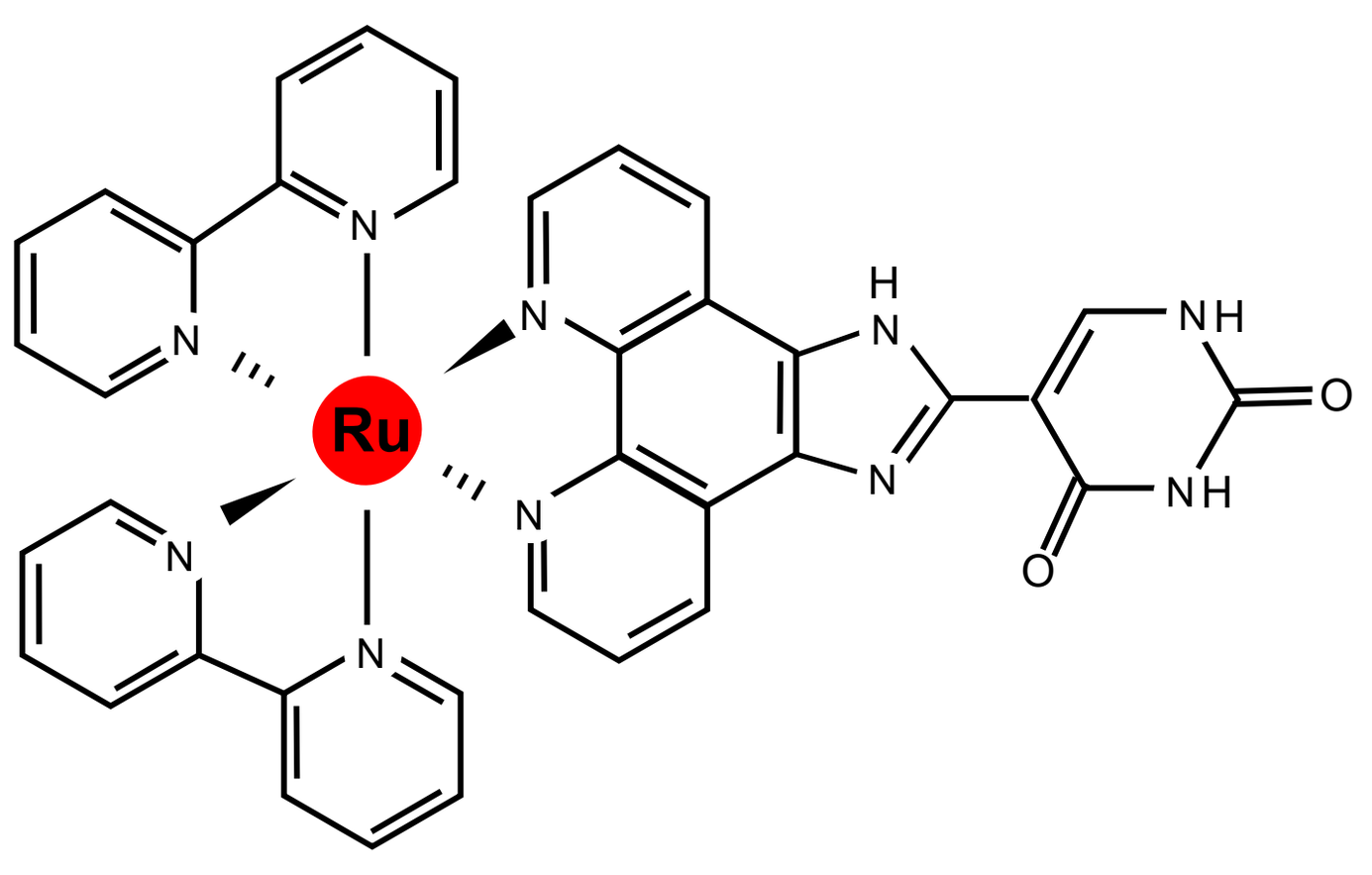

[Ru(bpy)$_2$(uip)]$^{2+}$ (Ru1)

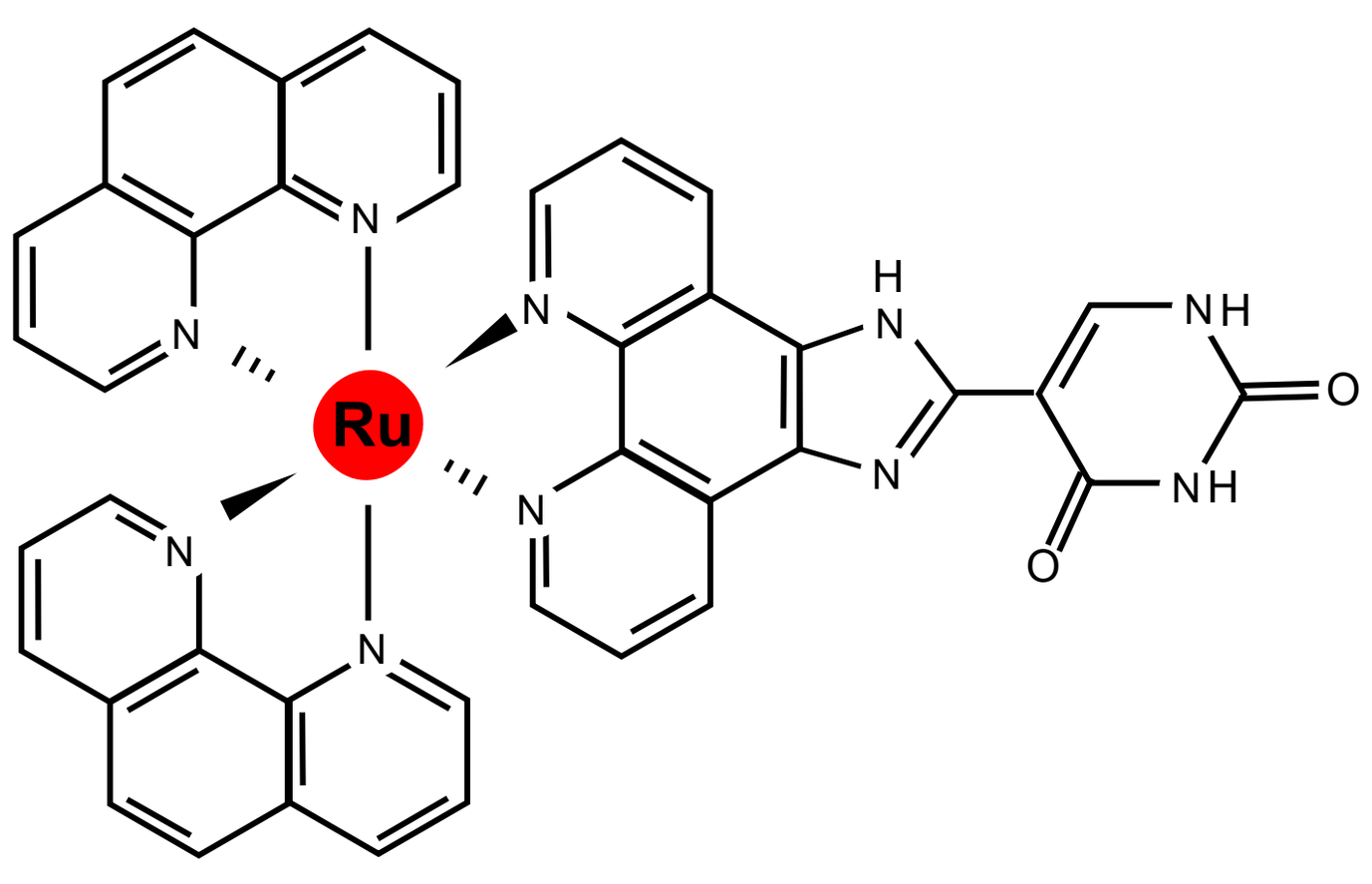

[Ru(phen)$_2$(uip)]$^{2+}$ (Ru2)

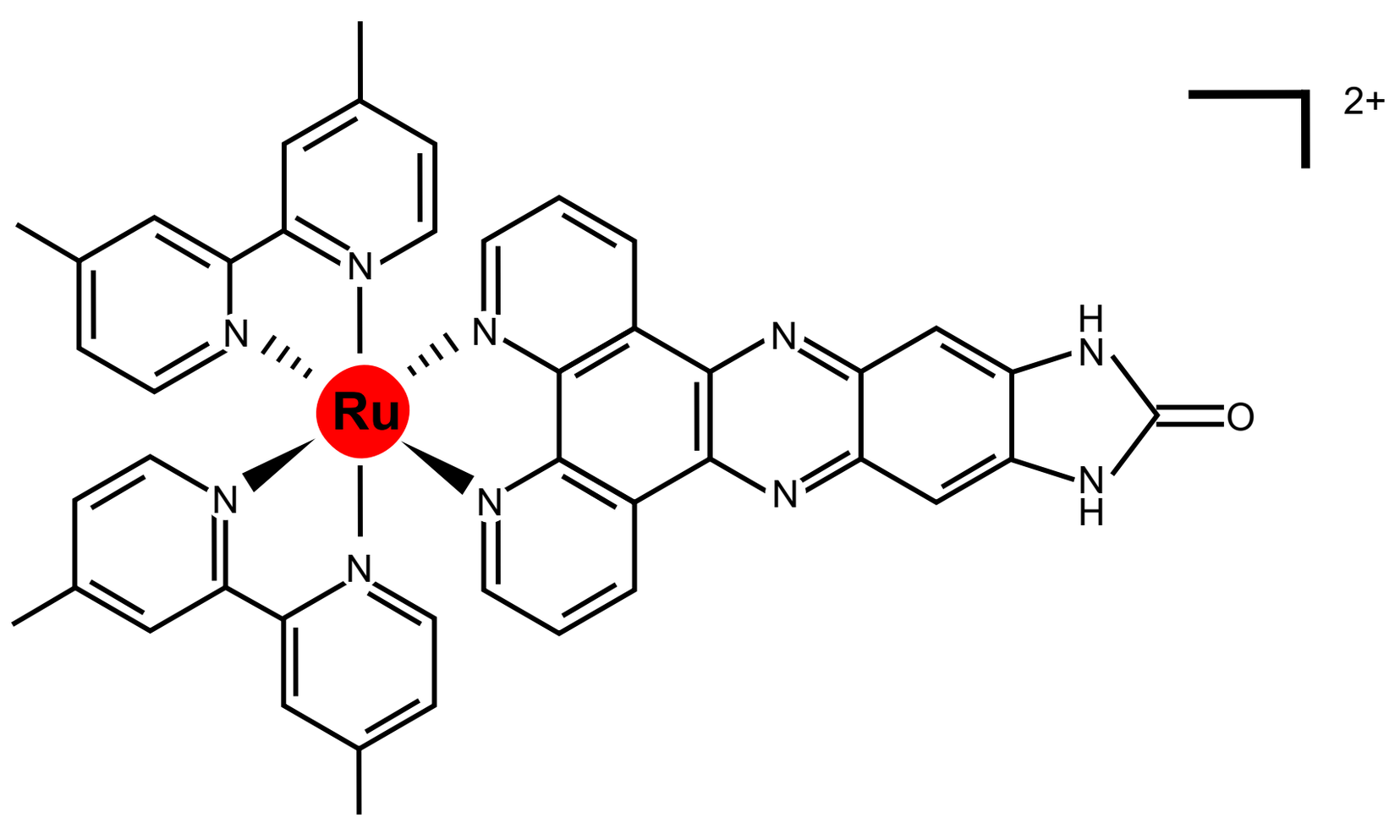

[Ru(dmb)$_2$(dppz-idzo)]$^{2+}$

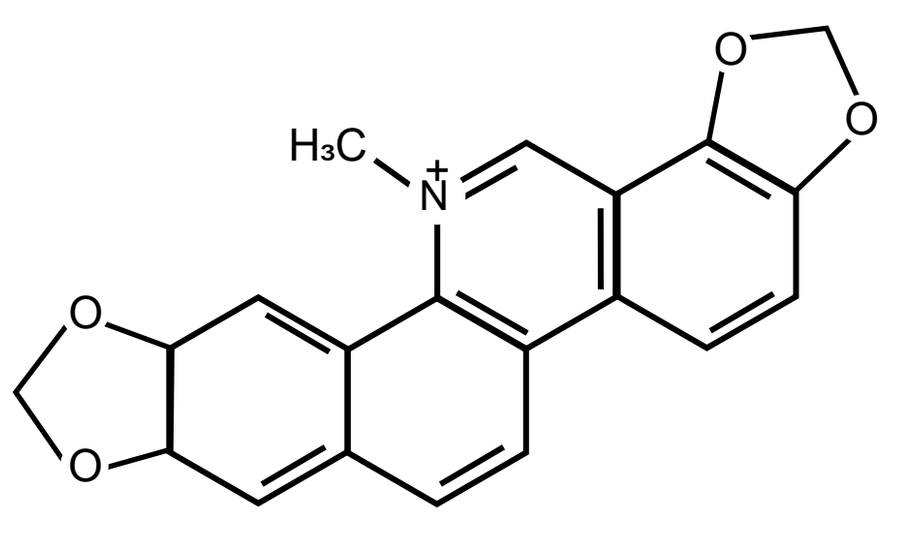

Sanguinarine

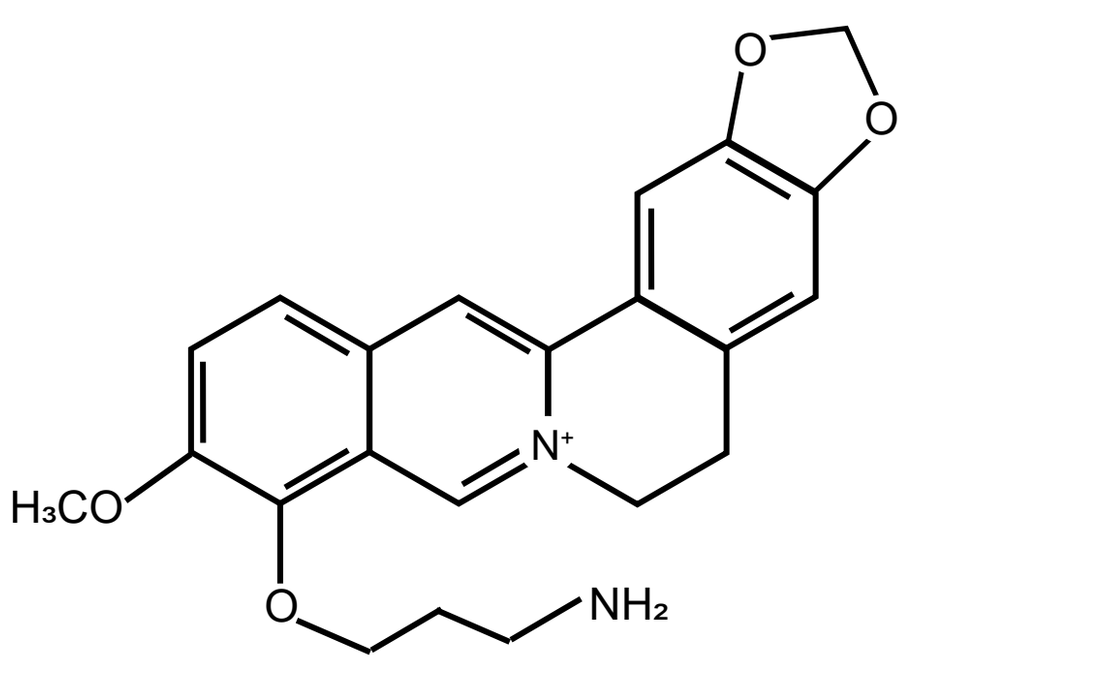

9-amino alkyl berberine analog BC1

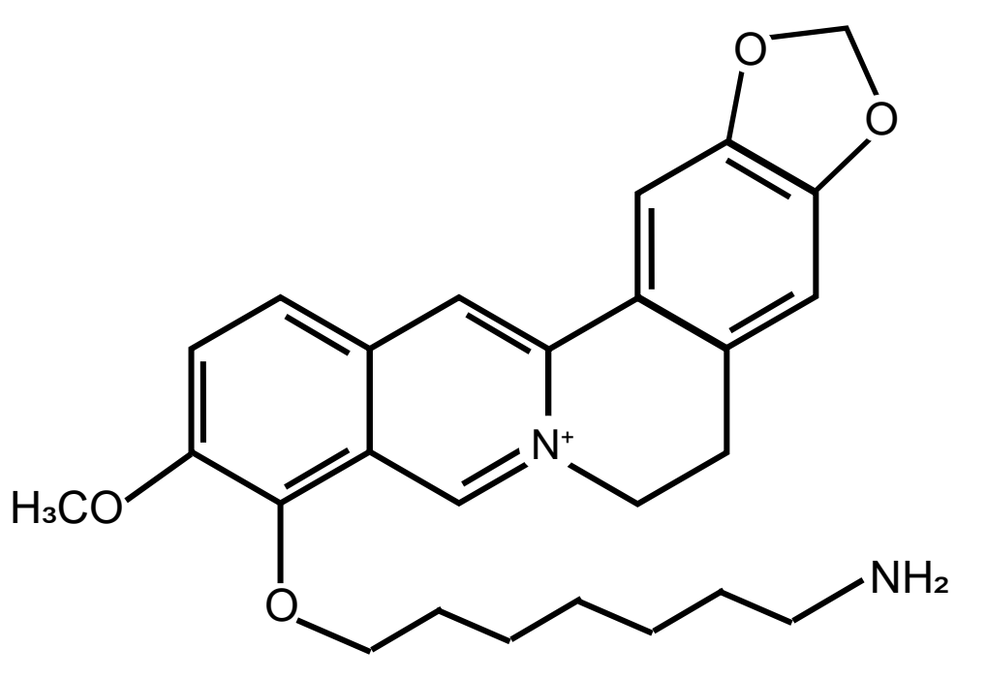

9-amino alkyl berberine analog BC2

Figure S1. Chemical structures of RNA triple-helix-targeting small molecules in Table 2. The chemical structures are ordered alphabetically.
